# Viral Communities Contribute More to the Lysis of Antibiotic-Resistant Bacteria than the Transduction of Antibiotic Resistance Genes in Anaerobic Digestion Revealed by Metagenomics

**DOI:** 10.1021/acs.est.3c07664

**Published:** 2024-01-24

**Authors:** Junya Zhang, Tiedong Lu, Yunpeng Song, Ulisses Nunes da Rocha, Jibao Liu, Marcell Nikolausz, Yuansong Wei, Hans Hermann Richnow

**Affiliations:** † State Key Joint Laboratory of Environmental Simulation and Pollution Control, Research Center for Eco-Environmental Sciences, Chinese Academy of Sciences, Beijing 100085, China; ‡ Department of Isotope Biogeochemistry, Helmholtz Centre for Environmental Research–UFZ, Leipzig 04318, Germany; § Department of Environmental Microbiology, Helmholtz Centre for Environmental Research–UFZ, Leipzig 04318, Germany; ∥ University of Chinese Academy of Sciences, Beijing 100049, China; ⊥ Agricultural Resource and Environment Research Institute, Guangxi Academy of Agricultural Sciences/Guangxi Key Laborarory of Arable Lnad Conservation, Nanning 530007, Guangxi, China

**Keywords:** phages, anaerobic digestion, antibiotic resistance genes, viral community, metagenomics

## Abstract

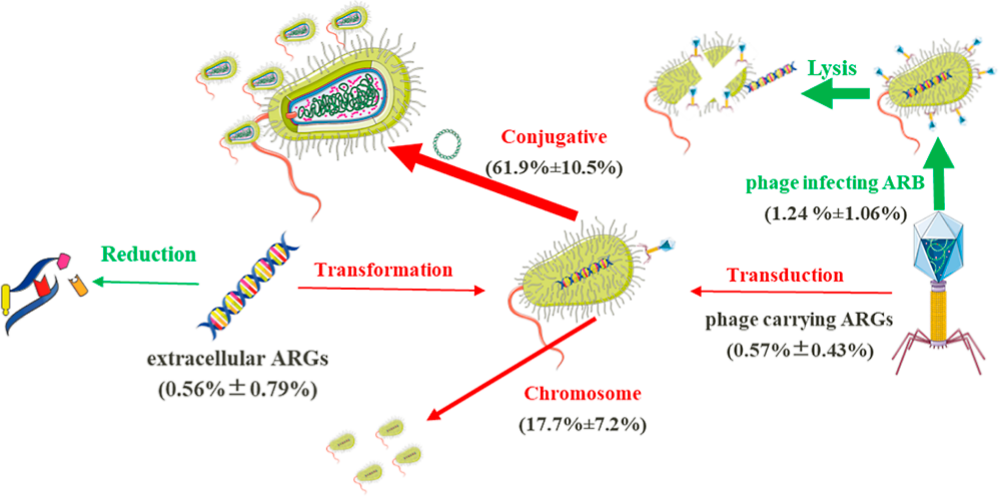

Ecological role of the viral community on the fate of antibiotic resistance genes (ARGs) (reduction vs proliferation) remains unclear in anaerobic digestion (AD). Metagenomics revealed a dominance of Siphoviridae and Podoviridae among 13,895 identified viral operational taxonomic units (vOTUs) within AD, and only 21 of the vOTUs carried ARGs, which only accounted for 0.57 ± 0.43% of AD antibiotic resistome. Conversely, ARGs locating on plasmids and integrative and conjugative elements accounted for above 61.0%, indicating a substantial potential for conjugation in driving horizontal gene transfer of ARGs within AD. Virus–host prediction based on CRISPR spacer, tRNA, and homology matches indicated that most viruses (80.2%) could not infect across genera. Among 480 high-quality metagenome assembly genomes, 95 carried ARGs and were considered as putative antibiotic-resistant bacteria (pARB). Furthermore, lytic phages of 66 pARBs were identified and devoid of ARGs, and virus/host abundance ratios with an average value of 71.7 indicated extensive viral activity and lysis. The infectivity of lytic phage was also elucidated through laboratory experiments concerning changes of the phage-to-host ratio, pH, and temperature. Although metagenomic evidence for dissemination of ARGs by phage transduction was found, the higher proportion of lytic phages infecting pARBs suggested that the viral community played a greater role in reducing ARB numbers than spreading ARGs in AD.

## Abstract 2

The microbiome and viral community in anaerobic digestion were analyzed with respect to their role to support or reduce proliferation of antibiotic resistance.

## Introduction

The increase in severe infections attributed to antibiotic-resistant bacteria (ARB) is a global issue, leading to higher mortality rates and escalating healthcare costs.^1,2^ According to the WHO report, nearly 700,000 deaths annually are closely associated with antibiotic resistance. This number will increase to 10 million by 2050 if no effective strategy is conducted to curb the dissemination of antibiotic resistance genes (ARGs).^3,4^ Within the One Health framework, the horizontal gene transfer (HGT) of ARGs between environmental microbes and human and animal pathogens is a critical concern.^5^

Three primary mechanisms drive HGT: transformation, conjugation, and transduction, and the important role of transformation and conjugation on the spread of ARGs has been extensively validated.^6−9^ Transduction mediated by lysogenic phages infecting bacteria and archaea is proposed as a significant HGT route capable of transferring a wide array of ARGs embedded in phages, although it is indicated that phages encoding ARGs are rarely found in human or mouse-associated environments.^10−13^ Nonetheless, lytic phages could serve as a solution to overcome antibiotic resistance in the near future through phage therapy.^2,14,15^ This is a dilemma: lysogenic phages are proposed to be crucial for ARGs’ dissemination in environmental contexts, yet phage therapy through the lytic phages is vital for treating ARB infections clinically.^16,17^ This leads to the urgent question of what precisely the phages do, considering the dual character of the phages (transduction vs host lysis) within the environmental context. Although viruses are the most numerous biological entities on earth, with an estimated population size of 10^31^ virus particles,^18^ the viral community has not been well investigated so far due to challenges in cultivating naturally occurring viruses. Moreover, viruses lack a universally conserved marker gene akin to the bacterial 16S rRNA gene, impeding their detection and classification like bacteria and archaea.^19^ Nonetheless, the rapid development of virus data sets and bioinformatics tools for mining viral sequences has enabled the investigation of viral community without experimental virus concentration and purification.^20−22^

In the environment, livestock manure and sewage sludge were the two major sources of ARGs due to the widespread use of antibiotics in the livestock industry and human healthcare system.^23,24^ Anaerobic digestion (AD), a crucial resource utilization technique for treating livestock manure and sewage sludge, has become a focal point in environmental engineering.^25−27^ Beyond its role in methane production for energy, AD plays a pivotal role in reducing ARGs and mitigating the presence of human pathogens to ensure biosafety. Numerous studies have consistently shown that the dynamics of microbial communities play a central role in shaping the fate of ARGs within AD systems.^28,29^ These changes are often attributed to vertical gene transfer (VGT), involving the proliferation or decline of the ARG’s hosts. However, the HGT network in AD remains poorly understood. While conjugation has been considered the primary mechanism driving the spread of ARGs, the potential influence of phage-mediated transduction should not be underestimated. Moreover, how phages impact the distribution of ARGs in AD needs further investigation. Consequently, clarifying the role of phage transduction vs phage therapy on the proliferation vs reduction of ARGs would help to control the ARGs within AD systems.

Here, we studied the role of viral community on the fate of ARGs in AD considering substrate types [pig manure (PM), chicken manure (CM), and sewage sludge (SS)], the microbiome of the substrate (with or without autoclaving), the inoculum sludge (accumulated or not), and the substrate organics (starch, proteins, and fatty acids). We aimed to (1) clarify antibiotic resistome-viral community relationship; (2) determine the abundance and diversity of viruses carrying ARGs; (3) establish the host-infection relationship between ARB and phages; and (4) compare the prevalence of lytic vs ARG-encoding phages within the AD systems. The outcomes of this study are expected to provide a fresh perspective on controlling the spread of ARGs within AD processes.

## Materials and Methods

### Experimental Setup

Three batch experiments of AD were established using the Automatic Methane Potential Test System II (Bioprocess Control, Sweden) in which digesters were a series of serum bottles (working volume: 0.4 L) equipped with a sampling tap and plastic caps including agitators and rubber stoppers. Three typical environmentally relevant ARG reservoirs were selected: (a) PM, (b) CM, and (c) SS from municipal wastewater treatment plants. In order to elucidate the role of the substrate microbial community on the fate of ARGs, experiments with the thermoautoclaved substrates (TPM, TCM, and TSS) were prepared through sterilization under 120 °C, 121 MPa, and 30 min. The validation of the autoclave effectiveness was confirmed through the R2A culture method, where no microbes could be cultured after the thermo-autoclave treatment.

The experiment design is summarized in Table S1. Briefly, for the batch experiment 1, the inoculum sludge was taken from a molasses wastewater treatment plant (nonacclimated sludge, NA). The microbes in this sludge have never been exposed to any of the three aforementioned substrates. For batch experiment 2, the inoculum sludge for each substrate type (acclimated sludge, A) was recovered from the respective anaerobic digestor treating the respective substrate. For batch experiment 3, the artificial substrate composed of starch, bovine serum albumin (BSA), and palmitic acid (PA) was used to represent the polysaccharides, proteins, and fatty acids, respectively, and the inoculum sludge was from the anaerobic digestor treating the sewage sludge. We considered the samples from the batch experiment 3 as the CK group because no ARGs were found in the artificial substrate when comparted to PM, CM, and SS. Five treatments covering the control (no substrate), M1 (starch/BSA/PA, 1:1:1, w), M2 (starch/BSA/PA, 4:1:1, w), M3 (starch/BSA/PA, 1:4:1, w), and M4 (starch/BSA/PA, 1:1:4, w) were established to represent the substrates with none, balanced, high polysaccharide, high protein, and high fatty acid content, respectively. Two mL of samples were taken directly from each bottle’s sampling aperture according to the methane production status covering the initial stage, intermediate stage, and end of the AD process for further analysis.

### DNA Extraction and Metagenomic Sequencing

The FastDNA Spin kit for soil (MP Bio, USA) was used to extract the total DNA from all the samples following the manufacturer’s protocols. The detailed information on metagenomic sequencing is shown in Supporting Information (Additional file 1). Briefly, a total of 59 sequencing libraries were established, and approximately 788 Gb of raw data were generated, which was trimmed and quality controlled through the metaWRAP-Read_qc module.^30^ The clean data were deposited in NCBI with the accession number of PRJNA863608.

The intracellular DNA and extracellular DNA of the samples from batch experiment 2 were separately extracted and used for (1) the conventional qPCR of selected ARGs to elucidate the results from metagenomics and (2) the comparison between intracellular ARGs (iARGs) and extracellular ARGs (eARGs). The detailed procedure for the extraction of iDNA and eDNA is shown in the Supporting Information.

### Profiling of the Antibiotic Resistome at the Read Level

The clean reads were blasted against the protein database of the Comprehensive Antibiotic Resistance Database (CARD, v3.1.4) through Diamond (v2.0.14, −query-cover 75, −id 90, −*e*-value 1 × 10^–5^) to determine the antibiotic resistome.^31,32^ The ARGs with risk level of rank I (current threats to human) and rank II (future threats to human) were identified through arg_ranker.^33^ In order to elucidate the results from the metagenomics, we quantified ARGs of *bla*_CTX-M_, *bla*_TEM_, *ereA*, *ermB*, *ermF*, *mcr*-*1*, *mefA*/*E*, *sul1*, *sul2*, *tetG*, *tetM*, and *tetX* along with *intI1* and 16S rRNA through the conventional qPCR for the iDNA and eDNA samples. The detailed information about the qPCR is described in the Supporting Information.

### Identify the ARGs Associated with Conjugation at the Contig Level

Each sample’s clean reads were assembled using MEGAHIT (v1.1.3, -mini-contig-len 1000) individually.^34^ The open read frames (ORFs) were predicted using Prodigal v2.6.3 (-meta) and then searched against the database of CARD (−query-cover 70, −id 80, −*e*-value 1 × 10^–5^). The ORFs located on the contigs carrying ARGs (ARCs) were further blasted against the ICEberg (integrative and conjugative elements) database.^35^ Meanwhile, the ARCs belonging to chromosomes or plasmids were determined through PlasFlow v1.1.^36^ The plasmids and ICEs were the two major elements capable of intercellular transfer of ARGs through conjugation, while other mobile genetic elements (MGEs) like insertion sequences (IS), transposons, and integrons depended on the plasmids and ICEs for the intercellular transfer.^37^ Thus, we considered the ARGs located together with plasmids or ICEs as potential conjugative mobility. Furthermore, the ARCs were taxonomically classified through the Taxator-tk (v1.3.3) and CAT.^38,39^ The relative abundance of ARCs was determined through coverM.

### Viral Contigs Identification, Clustering, and Taxonomic Assignment

The contigs >5.0 kb were collected, dereplicated and then piped through VirSorter2 (based on sequence similarity and other viral-like features such as GC skew) and VirFinder (based on *k*-mer signatures) for the identification of viral sequences.^40,41^ The identified viral contigs from VirSorter2 and VirFinder were merged and dereplicated with CD-HIT v4.7 at a local identity of 100%. The valid 21,518 viral contigs were subjected to species-level clustering to create viral operational taxonomic units (vOTUs) using the ClusterGenomes scripts, following the MIUViG recommended criteria of 95% average nucleotide identity (ANI) and 85% alignment fraction (AF),^42^ resulting in the identification of 13,895 vOTUs. Taxonomic assignment of these vOTUs was carried out using four methods (using the pre-2022 naming conventions): (1) vConTACT2;^43^ (2) majority-rules approach;^22,44^ (3) the blastn against Integrated Microbial Genome/Virus (IMG/VR, v3.0) and RefSeq virus database (release 203); and (4) CAT. Ultimately, 7767 of 13,895 vOTUs (55.9%) could be assigned to a taxonomic family. The detailed information is shown in Supporting Information (Additional file 1).

The phage lifestyle was further determined through VIBRANT (v1.2.1). To verify the novelty of vOTUs, the predicted ORFs were also compared to the IMG/VR v3 and RefSeq viral protein database using DIAMOND (−id 30, −query-cover 50, −*e*-value 1 × 10^–5^).

### Identify the Putative ARBs and Pathogens at the MAG Level

Metagenome assembly genomes (MAGs) were collected as follows: (1) by binning using metaBAT2, MaxBin2, and CONCOCT; (2) by refining the bins using Bin_refinement module in the MetaWRAP (v1.3.0) with completeness >70% and contamination <5%; and (3) by combining and dereplicating the refined bins through dRep.^45^ The dereplicated MAGs were taxonomically classified by GTDB-Tk (v1.5.0).^46^ Meanwhile, MAGs carrying ARGs were identified and termed as putative ARB (pARB), and MAGs carrying the virulence factor genes (VFGs) were identified through Victors database^47^ and treated as putative human pathogens.

### Virus–Host Interaction Analysis

The virus–host interaction was established based on CRISPR spacer, tRNA, and homology match method between viral contigs and MAGs.^48^ The CRISPR spacers in MAGs were collected through CRISPR Recognition Tool (CRT) using the default parameters,^49^ and tRNA were recovered from vOTUs by ARAGORN (v1.2.38).^50^ The vOTUs were searched against the curated CRISPR spacer database using blastn-short with 97% identity and 90% coverage.^33^ The tRNA sequences from vOTUs were blasted against MAGs with the parameters of 100% identity and 100% coverage without self-hits and duplicates. As for the homology match method, the best hits below an *e*-value threshold of 10^–5^ were considered a match when phages aligned with more than 80% sequence identity over a length between 1 kb and 50% of the microbial host contig.^48^

### Laboratory Test of Lytic Infectivity of Phages to ARB within AD

In order to testify to the infection of lytic phages to the ARB in the AD system, *Escherichia coli* BL21 (DE3) showing resistance to kanamycin on the plasmid pET28a was termed the ARB for the isolation of lytic phages. Seven lytic phages were isolated from the anaerobic sludge. Then, the infectivity was tested under the anaerobic condition, and how the changes of the phage-bacteria-ratio, pH, and temperature impacted the infection effectiveness was further determined. The detailed information is shown in Supporting Information (Additional file 1).

## Results and Discussion

### Substrate Types Significantly Impacted the Antibiotic Resistome in AD

One-way PERMANOVA based on Bray–Curtis revealed significant variability in the antibiotic resistome within AD across different substrate types (*p* < 0.05). In AD of PM and CM, the predominant classes of ARGs were associated with tetracycline resistance (35.7 and 35.9%, respectively), followed by macrolide-lincosamide-streptogramin (MLS) and aminoglycoside resistance, accounting for 28.4 and 30.2%, as well as 27.8 and 19.8%, respectively. Nonetheless, a higher abundance of sulfonamide resistance genes in AD of SS (19.3%) contributed the most to the difference from PM and CM (Figures S1 and S2). Interestingly, the CK, despite lacking microbes and ARGs within the artificial substrate, exhibited a notable presence of chloramphenicol resistance genes (6.3%).

**Figure 1:**
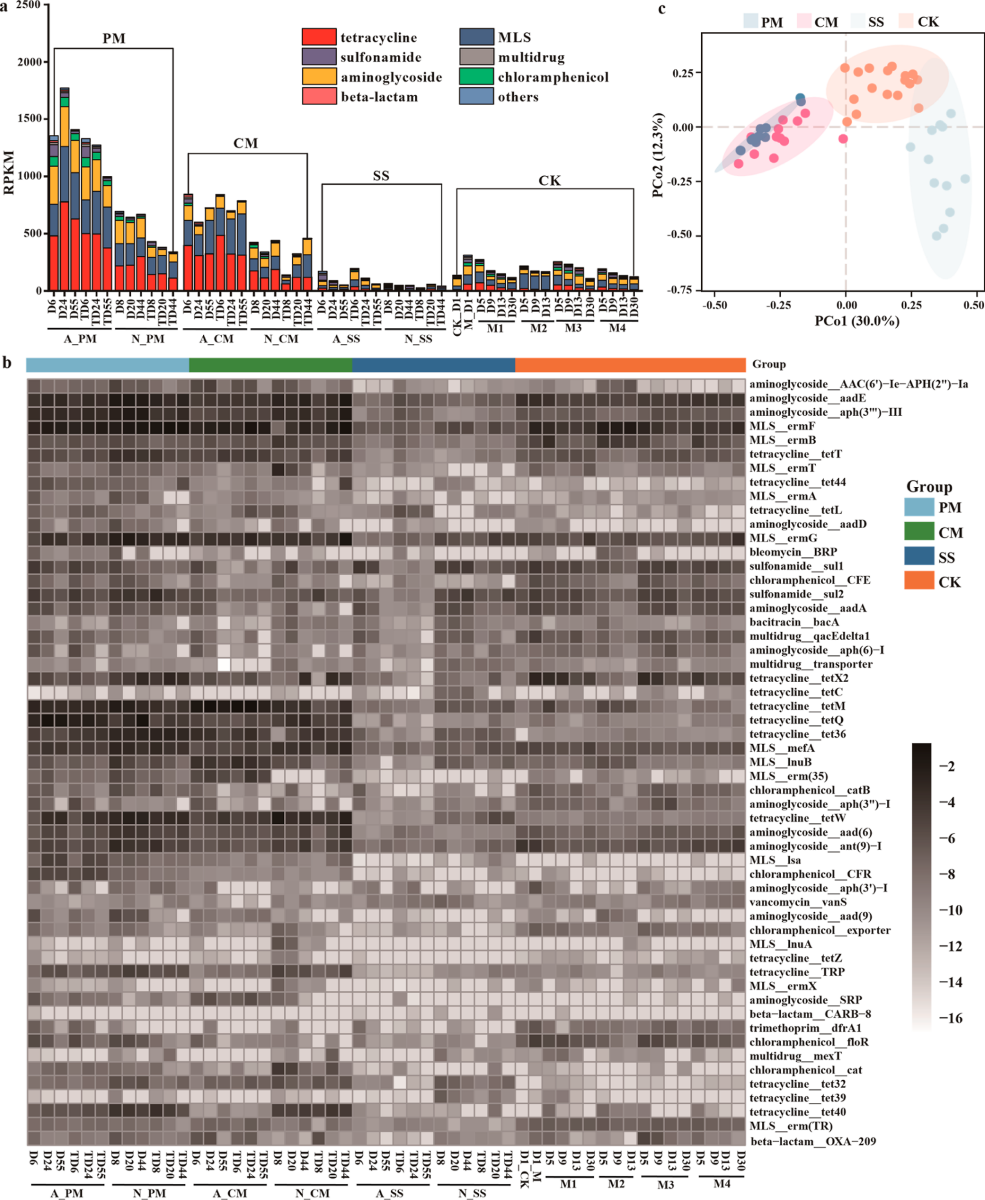
Changes of the ARGs’ classes in AD with various substrates (a); heatmap showing the dynamical changes of the top 10 ARGs in each sample (b), with the values indicating the relative abundance by log 2 transformed; and principal coordinate analysis (PCoA) of the antibiotic resistome based on the relative abundance of ARGs (c). PM: pig manure; CM: chicken manure; SS: sewage sludge; and CK: artificial substrates.

The relative abundance of ARGs in AD of PM and CM was observed to be 9.4 and 7.6 times higher, respectively, compared to SS (*p* < 0.01). Surprisingly, even in the CK lacking any microbes or ARGs in the substrate, an increase in the relative abundance of ARGs was noted (Figure 1a). Then, we determined whether inoculum sludge that was accumulated would impact the fate of ARGs. We found that relative abundance of ARGs with accumulated inoculum sludge was much higher than that with nonaccumulated (*p* < 0.05), indicating that long-term operation with substrates carrying amounts of ARGs could increase the relative abundance of ARGs in the sludge phase. Additionally, we conducted substrate sterilization through autoclaving to eliminate the presence of active microbes while preserving their organic composition. Our results indicated that substrate autoclaving can support ARGs’ reduction only to very limited extent in AD. In some cases, such as AD of CM with nonaccumulated inoculum sludge, it even resulted in a significant increase in the relative abundance of ARGs. These findings collectively suggested that the presence of living microbes in these substrates had minimal influence on the fate of ARGs within the AD system.

Moreover, we delved into the impact of the substrate organic composition on the fate of ARGs. The artificial substrates initially triggered an increase in the relative abundance of ARGs, followed by a decline throughout AD until reaching the initial level by the end of the process. In contrast, M3 group, characterized by high protein content, displayed a significant reduction in ARGs (Figure 1a). The composition of the antibiotic resistome changed significantly for M2 with high starch, where the MLS resistance genes were vastly enriched. The M2 and M4 with high fatty acids both showed a relative increase of ARGs compared to other groups. These observations underscored the fact that in comparison to the limited influence of living microbes in the substrate, the organic composition of the substrates could exert a more pronounced impact on the fate of ARGs.

The typical ARGs exhibited dominance in the AD of livestock manure like *aadE*, *aad*(*6*), *aph*(*3*‴)-*III*, *tetT*, *tetM*,and so forth, whereas *sul1* and *sul2* were prevalent in SS (Figure 1b). Nonetheless, MLS_*ermF* and aminoglycoside_*aadE* emerged as major ARGs common to AD. PCoA analysis of ARG types also indicated the significant difference between livestock manure (PM and CM) and SS along with CK (*p* < 0.05, Figure 1c). In relation to specific ARGs, conflicting trends have often emerged across different substrate types. For instance, *sul1* and *sul2* could be effectively reduced in the AD of SS with the accumulated sludge but enriched or changed little in the AD of livestock manure. Similar observations were made for *ermF*. The results from qPCR further confirmed this phenomenon (Figure S3 and Table S2). The individual eARGs were generally reported to be 2–3 orders of magnitude lower than iARGs,^51,52^ which was in accordance with results in this study. The AD of SS contained a much higher ratio of eARGs (7.89 ± 9.17%) compared to AD of livestock manure (1.69 ± 3.26%). Nonetheless, the total eARGs constituted a minor fraction of antibiotic resistome in AD (0.56 ± 0.79%), indicating the limited role of transformation in the dissemination of ARGs (Figure S4), although the transformation of eARGs into potential pathogens has been reported.^53^ However, the ratio of eARGs increased significantly at the end of AD (10.16 ± 8.27%), where bacterial consortia may enter the decline phase, and the death rate may overtake the growth rate due to nutrient depletion.

The prevailing high-risk ARGs belonged to MLS_*ermT* (AAX84025) and MLS_*lnuB* (AAL05554) in AD across various substrates, even the CK (Figure S5 and Table S3). The two dominant ARGs were enriched in human-associated environments by 470 times and 239 times, respectively, with gene mobility of plasmid and the presence in the WHO ESKAPE pathogens.^33^ While the high-risk ARGs only accounted for 3.6 ± 2.2% of the total ARGs in AD, and no matter what kind of conditions, AD effectively facilitated their reduction, underscoring the pivotal role of AD technology in curtailing the propagation of high-risk ARGs within the environment.

### Antibiotic Resistome Exhibited Strong Conjugative Mobility in AD

**Figure 2:**
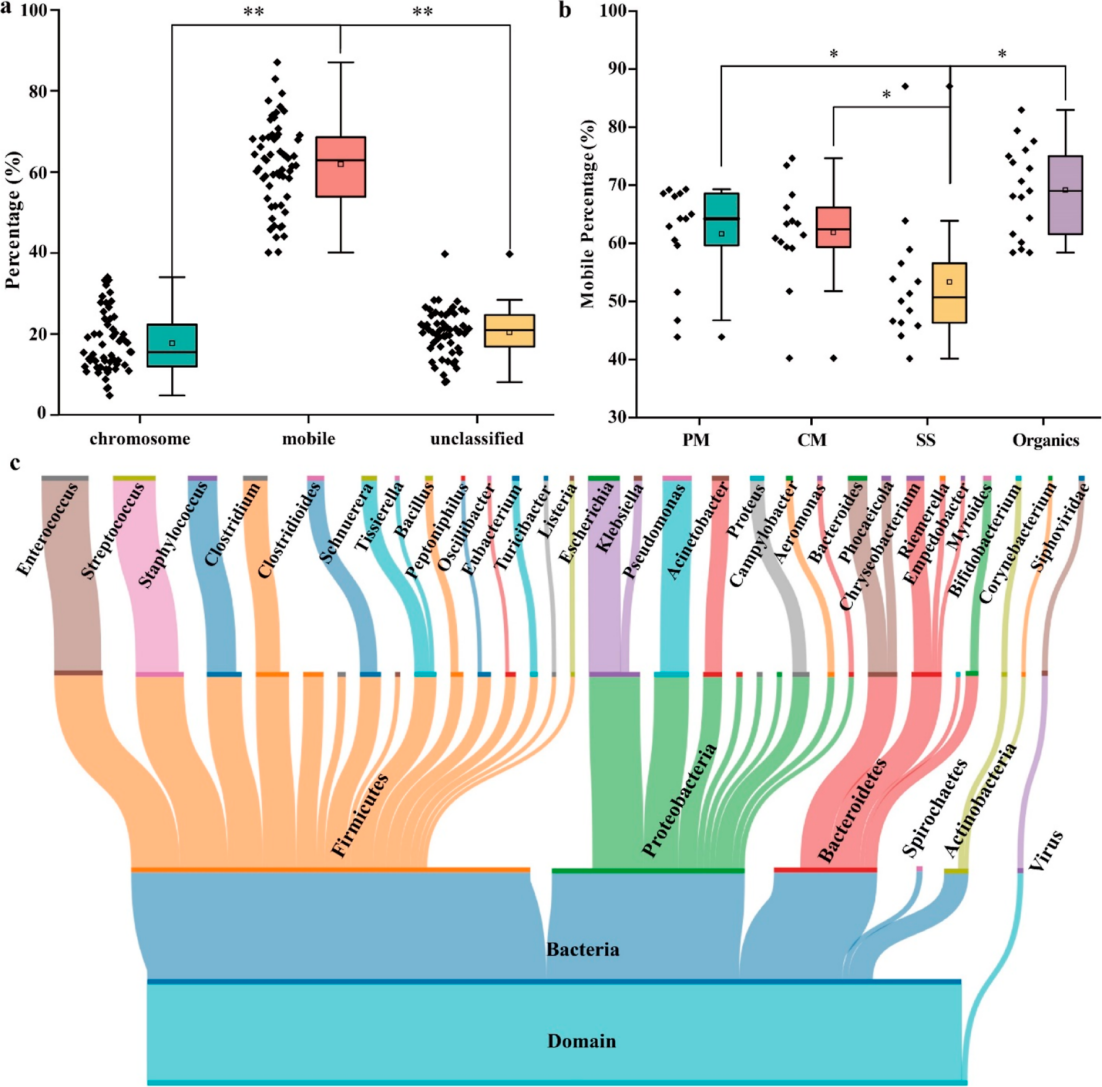
Percentage of chromosome carrying and mobile ARGs in AD (a); comparison of the mobile ARGs among substrate types in AD (b); and overall distribution of the taxonomic classification of the ARCs in AD (c). PM: pig manure; CM: chicken manure; SS: sewage sludge; and CK: artificial substrates.

We collected 3269 ARCs from 3,297,768 assembled contigs, where 555 of them were located on chromosome and 1989 on plasmids or ICEs (Tables S4 and S5). This indicated that 60.8% of the ARCs exhibited potential for conjugative mobility, with the value reaching 61.9 ± 10.5% in terms of abundance (Figures 2a and S7). These findings were comparable with the pattern found in combined sewage overflows (50.7%) and samples from municipal wastewater treatment plants (55.0%).^54,55^ Considering the 268 ARCs carrying high-risk ARGs, 236 of them (88.1%) were conjugatively mobile. The conjugative mobile ARCs also varied with substrate types, with AD of SS having the fewest mobile ARCs. The percentage of mobile ARCs in AD of livestock manure (PM and CM, 61.7%) was much higher than that in SS (53.3%), while CK had the highest percentage of mobile ARGs (69.1%, Figure 2b). Conjugation is widely recognized as an ATP-driven process, and the easy degradation of the artificial substrates could provide enough ATP. The nutritional aspect, as inferred from the soluble chemical oxygen demand and volatile organic solids, along with selective pressures stemming from antibiotic concentrations, were much higher in AD of livestock manure than those in SS as indicated in our previous study.^27^ This interpreted the higher proportion of conjugative mobility within the AD of PM and CM. Nonetheless, the highest proportion of conjugative mobility of ARGs in the CK without antibiotics indicated that nutrition played a more critical role in the conjugation than selective pressures.

### Prominent Role of VGT and Noteworthy Contribution of HGT to Antibiotic Resistome

**Figure 3:**
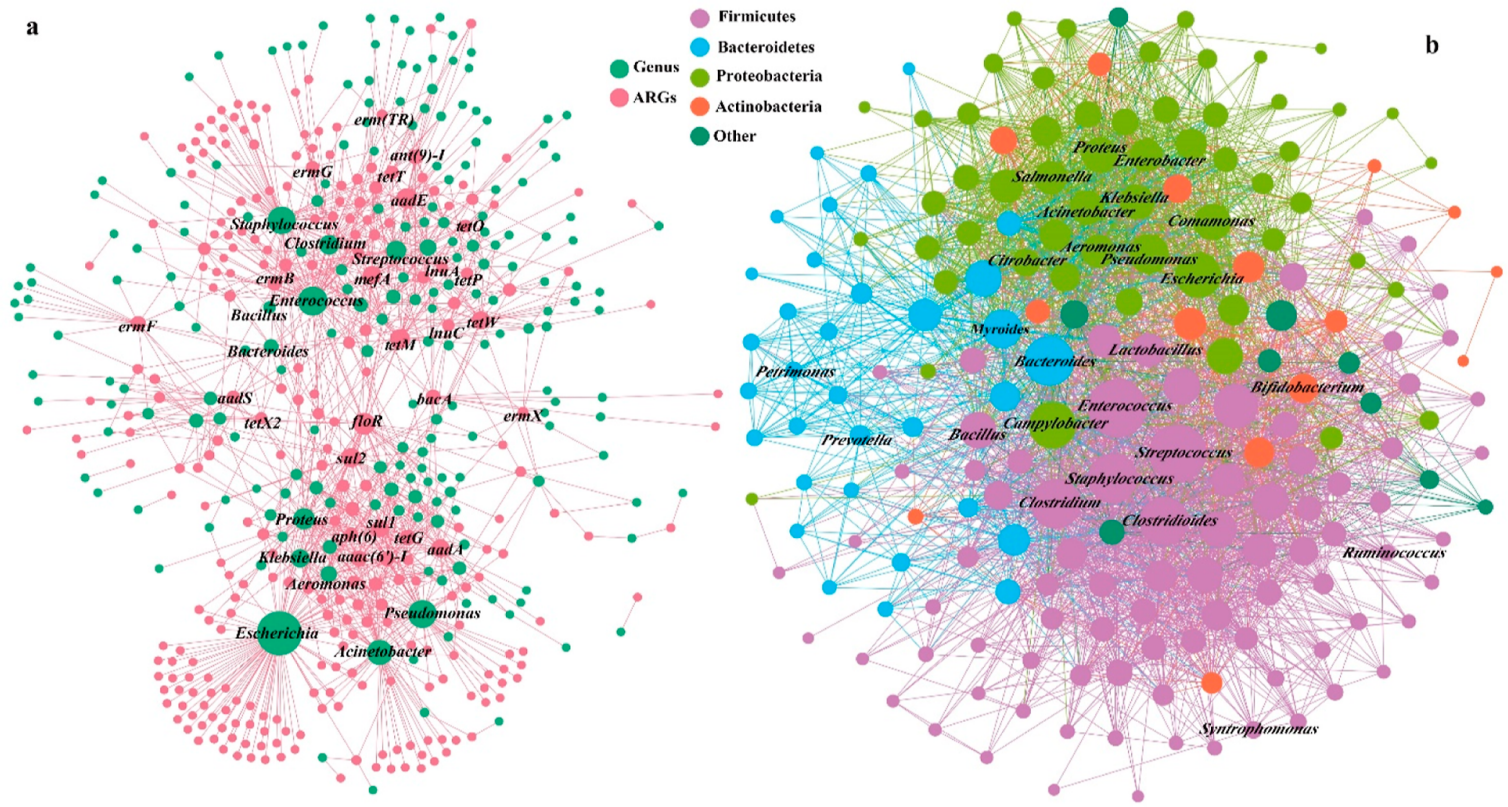
VGT network (a) showing the potential hosts of ARGs and HGT network (b) showing the genus (nodes) connected by at least one observed HGT event (edges). The genus was colored according to the taxonomy at the phylum level.

The dominant phylum of the ARG hosts were Firmicutes, Proteobacteria, and Bacteroidetes (49.0, 23.7, and 12.7%, respectively, Figure 2c), which is in accordance with their prevalence in the AD system.^56^ Although some ARCs could be reliably assigned to the species level, we considered the host information at the genera level to make the results more stringent. Among the 3269 ARCs, 1916 could be assigned to genera, forming the basis for establishing the VGT network depicting the interplay between ARGs and their hosts (Figure 3a). The genus *Escherichia* harbored the most diverse ARGs (91) followed by *Enterococcus* (53), *Pseudomonas* (50), *Staphylococcus* (49), and *Acinetobacter* (43) in AD. These genera belonged to potential human pathogens and were considered as high-priority targets to be monitored by WHO.^57^ Furthermore, we identified several crucial functional genera related to methane production, such as *Syntrophomonas*, *Proteus*, and *Ruminococcus*, carrying ARGs.

Among the ARGs, *floR* exhibited the broadest host range (34 genera) followed by *aadE*, *tetM*, *sul2*, and *tetW*, illustrating their prevalence in AD (Figure 1b). Additionally, we delved into microbial community structure at the species level using assembled 16S rRNA sequences with an average length of 1458 bp. The methods and results are described in detail in Supporting Information (Additional file 1 and Table S6). The Mantel test revealed a significant correlation between microbial community composition and the antibiotic resistome (*p* < 0.01). Microbial community contributed the most to antibiotic resistome as we could observe in Procrustes analysis (Figure S8). The variances of microbial community composition at the species level could explain 65.2% of the difference of antibiotic resistome across samples. These highlighted the dominant role of changes of microbial community composition through VGT of ARGs on the fate of antibiotic resistome in AD.

We identified a total of 5942 HGT events occurring among microbes at the genus level within the AD (Figure 3b). The HGT network covered 195 different genera, and the link between *Escherichia*–*Pseudomonas*, *Escherichia*—*Proteus,* and *Enterococcus*–*Staphylococcus* shared 24 types of ARGs. *Enterococcus* and *Streptococcus* exhibited the highest number of HGT events with other genera, where *Enterococcus* engaged in HGT events with 135 genera, while *Streptococcus* interacted with 132 genera. On average, there were 34 HGT event links between different genera. These findings underscore the extensive range of HGT events associated with ARGs, a trend that aligns well with the high proportion of ARGs exhibiting conjugative mobility in the AD environment.

### Viral Community Significantly Correlated with the Bacterial Community in AD

The DNA sequence comparison revealed that 98.8% (13,722 out of 13,895) of the vOTUs were novel as they exhibited no homologous sequences in either the IMG/VR 3.0 or Refseq virus database (release 203). A total of 197,459 viral ORFs were predicted, of which 28.1% lacked any protein homologue in the IMG/VR 3.0 database (identity >30%), while 76.9% exhibited no protein homologue in the Refseq virus database. Although four methods were used for the taxonomy, there are still 44.1% of the vOTUs that cannot be assigned phylogenetically to a specific family (Table S7). These indicated that there were great amounts of unknown information waiting to be discovered for viral community in AD. According to CAT, most of vOTUs belonged to phages that infected both bacteria (10,986 vOTUs) and archaea (254 vOTUs), whereas only 10 vOTUs were identified as eukaryotic viruses. Importantly, human viruses were absent from our findings, and the host organisms associated with these eukaryotic viruses encompassed Opisthokonta, Amoebozoa, Metamonada, and Apusozoa. In the next step, the taxonomy and abundance of the phages were further analyzed.

**Figure 4:**
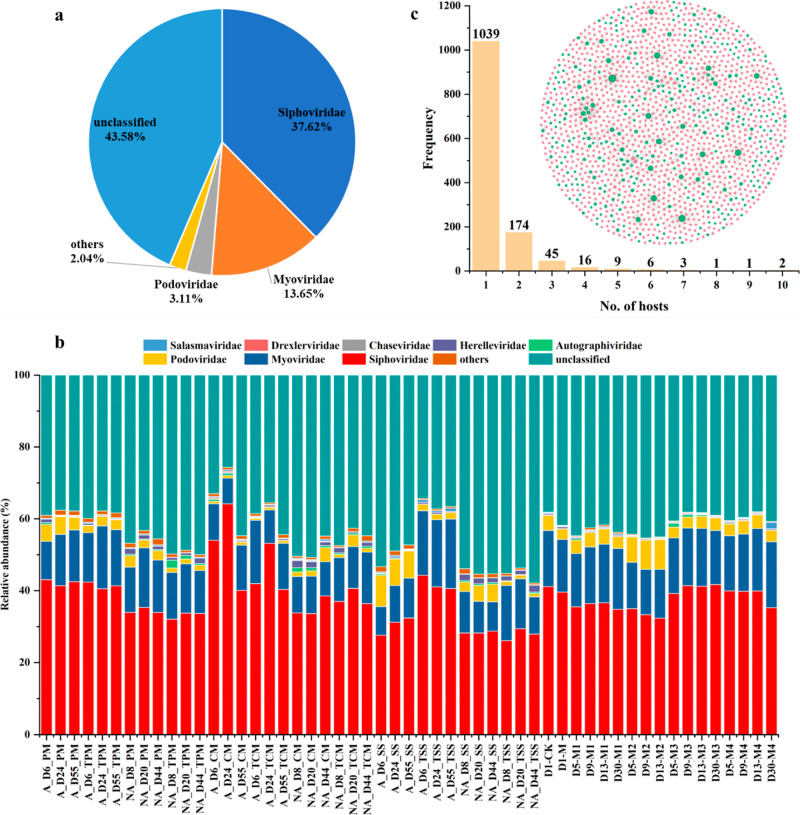
Composition changes of viral community in AD at the family level (a,b) and the frequency of the multiply hosts within AD based on the phage-host connection network (c).

Caudovirales belonging to Uroviricota emerged as the predominant virus, accounting for 55.9 ± 6.6% of the viral community in AD. Siphoviridae and Myoviridae were the two dominant phages at the family level, which accounted for 37.6 ± 6.8 and 13.7 ± 6.6%, respectively (Figure 4a). The two viral families have also been frequently observed to be abundant in the human gut viromes.^58^ A significant difference was observed between substrate types for viral community through the one-way PERMANOVA analysis and PCoA (Figures 4b and S9), analogous to the patterns observed in the microbial community (*p* < 0.05). The viral community also changed along with the AD. Whereas, the difference caused by the substrate microbiome and inoculum sludge was very limited compared to the substrate types. The Mantel test (*p* < 0.01) and Procrustes analysis indicated the substantial role of viral community on microbial community dynamics in AD (Figure S10). It was assumed that bacterial composition would shape the viral community and vice versa. Phages can infect and rapidly kill the bacterial host by multiplying inside and then bursting the bacterium to release the subsequent phage progeny, ready to infect and kill other nearby target bacterial cells.^59^ Nonetheless, when there were not enough target bacteria left for the phages to infect, the phages will also be reduced along with time in AD.

According to phage lifestyle analysis, our data revealed that most of phages (78.1 ± 5.9%) were lytic (Figure S11). The percentage of temperate viruses (21.9%) in AD was much higher compared to human gut (13.0%, GVD data set), soils (5.0%, IsoGenie data set), and oceans (3.0%, GOV2 data set).^22^ This discrepancy can be attributed to the fact that the databases for these data sets often focused on capturing viral-like particles (VLPs), potentially leading to an oversight in detecting temperate phages that have integrated into host genomes as prophages.^22^ The utilization of bulk metagenomics, as implemented in our study, enabled the detection of phages closely associated with bacteria and temperate phages that might otherwise be overlooked.

We collected 480 dereplicated high-quality MAGs (completeness >70%, contamination <5%). Although the coverage rates of these MAGs in PM, SS, and CM metagenomes were relatively low (16.9 ± 19.1%), they could cover 61.5 ± 11.1% of the entire microbiome in AD (Table S4). Within the AD system, we successfully established 2017 phage-host connections between vOTUs and MAGs using a combination of the CRISPR spacer, tRNA, and homology match method (Table S8). To ensure the reliability of these connections, we required that both the vOTU and MAG coexisted in at least one sample, with a minimum of 75% coverage of their bases in the clean reads. This stringent criterion yielded a collection of 1725 reliable phage-host connections within the AD system, where the host relationship between 1296 vOTUs and 336 of the 480 MAGs were established. It is worth noting that while a majority of vOTUs (60.2%) were primarily associated with a single species (MAG), a significant portion (39.8%) exhibited the potential to infect multiple genera, families, orders, and even classes (Table S9). These findings align with a survey of gut phages using meta3C proximity ligation, which reported that approximately 69.0% of gut phages were limited to infecting a single species.^60^ One of the most prevalent phage-host connections was observed in 40 out of 59 samples, involving a lytic phage and a MAG assigned as Petrimonas sp002356435. The two vOTUs that can infect 10 different MAGs belonged to Siphoviridae. Recent studies also indicated that broad-host-range phages were potentially widely distributed in the environments.^61^ Although these broad-host-range phages here were lysogenic, they did not carry any ARGs. Intriguingly, the MAGs assigned as g__JAAZFF0 (harboring five different ARGs), s__JAAYPV01 sp012523875, g__UBA3907 (containing two virulence factor genes), and g__Tissierella_B (with one virulence factor gene) demonstrated the highest diversity of phages capable of infecting them, with more than 30 associated vOTUs.

### Viral Community Played a Limited Role on the Dissemination of ARGs within AD

**Figure 5:**
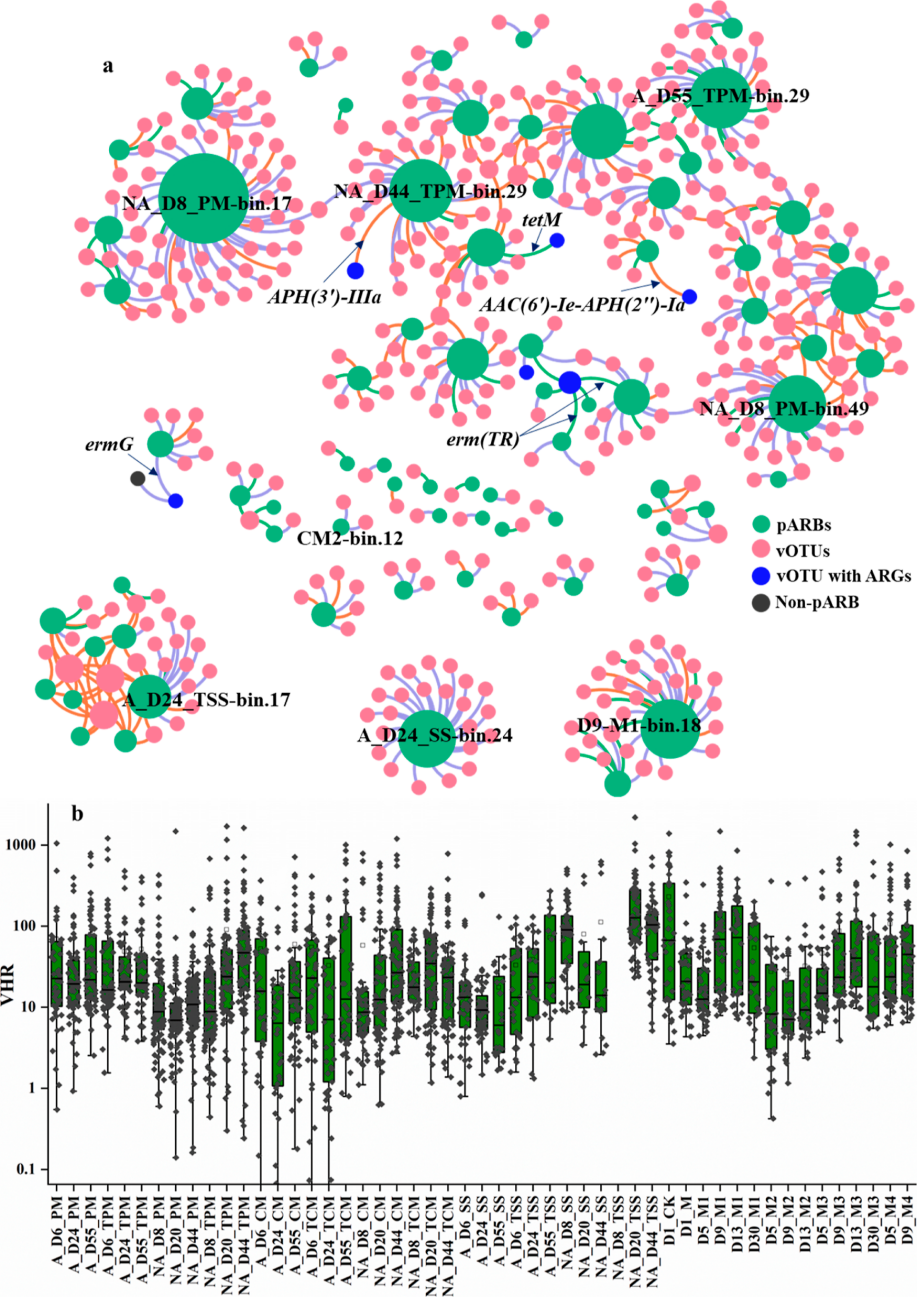
Network analysis showing the phage-host connections between viral community and potential antibiotic resistant bacteria (pARBs), and the purple, orange, and green line indicate that the connected vOTU belonged to the lytic, lysogenic, and undetermined lifestyle, respectively (a). Arrows indicate that the HGT event by the phage transduction happened, and the ARG was shared by the vOTU and its hosts; changes of virus/host abundance ratios (VHRs) for the viruses infecting the pARBs within AD (b). PM: pig manure; CM: chicken manure; SS: sewage sludge; and CK: artificial substrates.

We further discovered 21 vOTUs carrying ARGs (Table S10), and 2 of them were found to carry high-risk ARGs (rank I) of *mphB* and *APH*(*3*′)*-IIIa*. Nonetheless, virus carrying ARGs only accounted for 0.57 ± 0.43%, comprising a very small part of AD antibiotic resistome. Although the majority of these ARG-carrying vOTUs were lytic (11 out of 21 vOTUs), lysogenic phages might still play a role in the dissemination of ARGs in the AD system. Among the established virus–host information between 1296 vOTUs and 336 MAGs, 72.1% of the vOTUs were lytic, in accordance with the ratio of lytic phages within AD (78.1 ± 5.9%), while 6 vOTUs were carrying the ARGs, and 2 of them were found to be lysogenic. They shared the same ARGs with their hosts, which indicated that the phage transduction actually happened within AD (Figure 5a). Additionally, 13 out of the 21 vOTUs were identified as plasmid-phages or were associated with ICEs, which further increased the potential HGT of ARGs. Because the spread of ARGs by phage-plasmids could dispense cell-to-cell contact necessary for conjugation.^62^ However, considering the much higher abundance of conjugative mobile ARGs (61.9 ± 10.5 vs 0.57 ± 0.43%), the contribution of phage transduction to ARGs proliferation was likely limited. Furthermore, phages carrying ARGs exhibited relatively narrow host ranges, which further restricted the potential for HGT between phylogenetically distinct bacteria in the AD system (Figure 5a and Table S8). Although some studies considered the phages as a kind of reservoir of ARGs,^63−65^ the prevailing view among researchers considered that phages rarely encoded ARGs.^10,13,66^ We further confirmed that few of the phages in AD were carrying ARGs and elucidated the lower proportion of phages carrying ARGs in antibiotic resistome.

### Viral Community Played a Greater Role in the Reduction than Dissemination of ARGs within AD

In contrast to the proliferation of ARGs, we further recognized the phage-host relationship with ARBs to identify the potential role of phages in the reduction of ARGs in the AD system. Among the 480 MAGs, 95 was found to carry ARGs and treated as putative ARBs (pARBs), where 56 of the pARBs were found to carry VFGs and 12 with high-risk ARGs (Table S9). We established the host relationship between the viral community and 76 of the 95 pARBs (Table S11). Lytic phages were identified in connection with 66 of the 76 pARBs (Figure 5a). For instance, CM2-bin.12 classified as *g__Oceanobacillus* carried 15 types of ARGs, and we established the host relationship with 2 lytic vOTUs that did not carry any ARGs. A similar situation occurred with A_D24_TSS-bin.17, which carried both 15 virulence factor genes and multidrug_*rsmA*. These highlighted the role of phages in the control of pARBs in AD.

Among the 21 vOTUs carrying the ARGs, 6 were found to have the ability to infect the pARBs. Interestingly, five of these vOTUs shared the same ARGs with their corresponding pARB hosts, specifically involving the five ARGs: *erm*(*TR*), *tetM*, *ermG*, *APH*(*3*′)*-IIIa*, and *AAC*(*6*′)-*Ie*-*APH*(*2*″)-*Ia* (Figure 5a). For instance, it is possible that the high-risk ARG of *APH*(*3*′)*-IIIa* carried by NA_D44_TPM.bin.29, assigned as Fermentimonas sp002398875, originated from the lysogenic phage of the vOTU of A_D24_SS_248532_length_47810_cov_11.0114. These indicated that phage transduction indeed contributed to the dissemination of ARGs within AD. Nonetheless, 10 lytic vOTUs without ARGs were found to infect the same host. Alarmingly, vOTU with the identifier D5-M3_98818_length_96315_cov_22.9974, carrying the *erm*(*TR*), exhibited a notably broad host range, encompassing families such as Cellulosilyticaceae, Clostridiaceae, Sporanaerobacteraceae, Halanaerobiales, and Peptoniphilaceae. Disturbingly, the transfer of *erm*(*TR*) via transduction occurred within two of its host organisms, specifically in D5-M3-bin.2 and NA_D8_CM-bin.37 (Figure 5a). This highlighted the urgent need for vigilance regarding the potential dissemination of *erm*(*TR*) into other hosts within AD. Although six potential HGT connections through phage transduction were identified based on the phage-host analysis, we could find more lytic phages that could eliminate the pARBs. A total of 292 connections were established between lytic vOTUs and pARBs. These indicated that the viral community played a greater role in reducing ARGs compared to its role in disseminating them within AD.

### Infection of Viral Community to pARB Actively Happened within AD

It was assumed that if viruses had much higher abundance than their hosts, they should facilitate a very active viral replication and possible lysis, which could be reflected by virus–host ratios (VHRs).^20^ In this study, most of the VHRs were above 1.0, with an average value of 71.7 (Figure 5b). The mean VHRs in different ecosystems were reported to range from 5.6 to 704.4, and activated sludge and ocean had a similar value of 26.5 vs 26.2.^10,67^ These could indicate a high level of active viral infection of the pARBs within AD. For instance, the VHR between the lytic phage of NA_D8_PM_347733_length_67298_cov_154.7239 and the NA_D8_PM -bin.44 classified as Cellulosilyticaceae carrying the *vanG* and *vanRG* was as high as 1418.6, indicating high viral activity on lysis.

**Figure 6:**
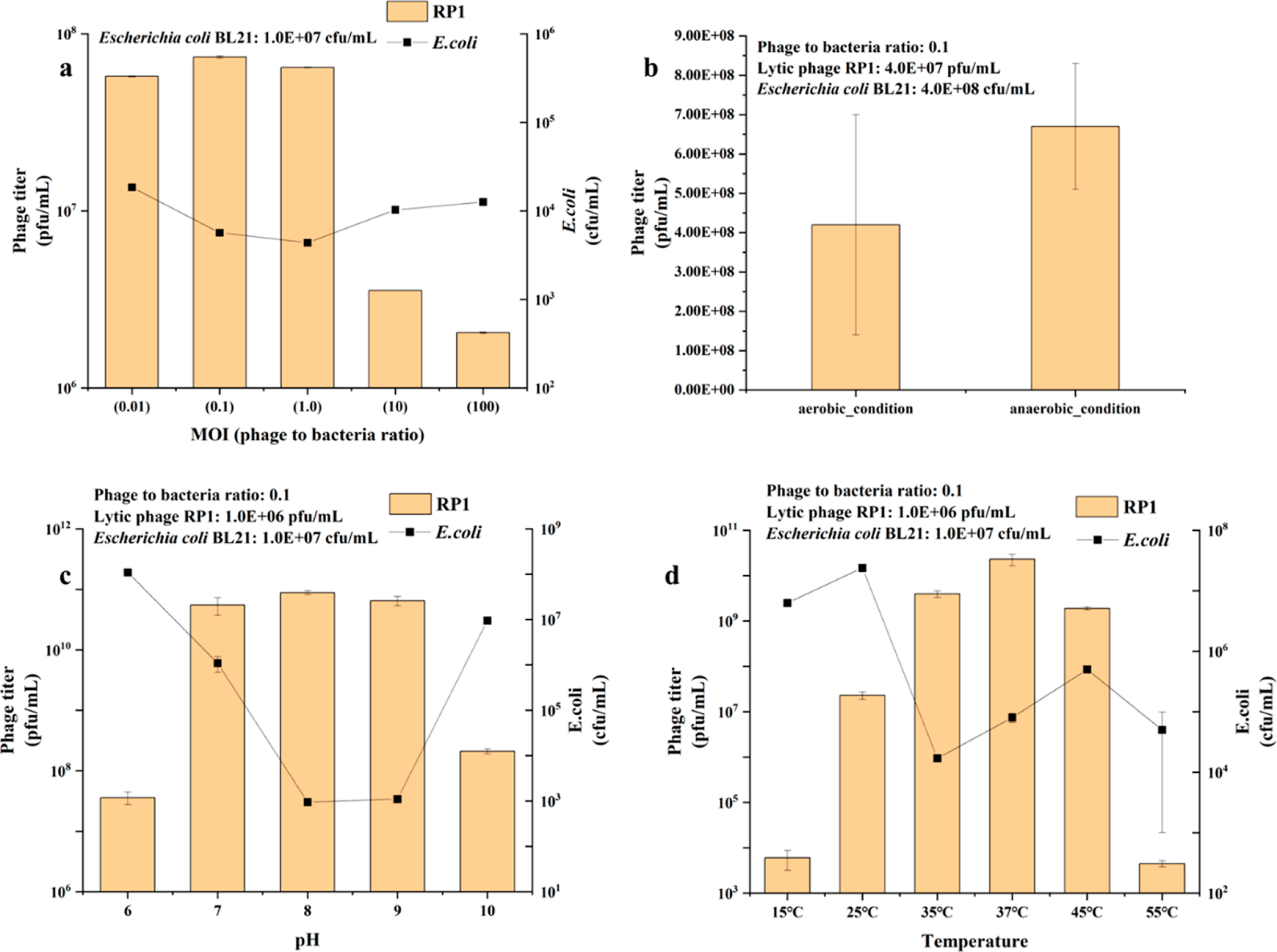
Effect of the phage-to-host ratio on the lytic infectivity to the host (a). Comparison of the phage titers between aerobic and anaerobic conditions (b). Effects of pH (c) and temperature (d) on the lytic infectivity under anaerobic condition. Error bars represent standard deviation (s.d.) of three sampling replicates (*n* = 3).

Furthermore, seven lytic phages capable of infecting *E. coliBL21* (*DE3*) with kanamycin resistance were isolated and purified from anaerobic sludge (see the Supporting Information). Their lytic infectivity under anaerobic conditions was confirmed, and it was observed that they produced larger phage plaques compared to those under aerobic conditions (Figure S12). Although the formation of clear phage plaques required a significantly longer time (7 days) under anaerobic conditions due to the much slower growth rate of the host bacteria compared to the aerobic condition (12 h), the phage concentration could reach higher maximum levels under anaerobic conditions (Figure 6). This indicated that phages might be more effective on the control of ARB within the AD system. Several factors were found to impact infectivity, including the phage-to-host ratio, pH, and temperature. Interestingly, the lytic phage RP1 showed a larger lytic effect under a phage-to-host ratio of 0.1, and a greater ratio of 10 and 100 did not enhance the lytic effect. The pH and temperature were also found to significantly affect the infectivity within AD. Alkaline conditions with a pH of 8–9 exhibited larger lytic effects as did mesophilic conditions. Conversely, low temperatures and thermophilic conditions greatly reduced the infectivity of the lytic phages. It should be noted that these results were specific to one lytic phage under anaerobic condition, and outcomes may differ with other lytic phages.^68^ Nevertheless, the significant impact of pH, temperature, and the phage-to-host ratio on the infectivity of lytic phages to control ARB within AD systems seems to be evident.

### Environmental Implications

In summary, the following results were collected for the phages associated with antibiotic resistome in AD: (1) conjugation was the primary way for the HGT within AD surpassing both eARGs’ transformation and phage transduction, and eARGs only constituted a small part of antibiotic resistome in AD (0.56 ± 0.79%), conjugative mobility ARGs accounted for 61.9 ± 10.5%, while it was 0.57 ± 0.43% for viruses carrying ARGs; (2) although six potential HGT connections through phage transduction were identified, a total of 292 connections were established between lytic vOTUs and pARBs; (3) among the 76 pARBs whose infecting phages were determined, 66 contained lytic phages; and (4) the abundance of phages infecting pARBs (1.24 ± 1.06%) was much higher than that of phages carrying ARGs (0.67 ± 0.85%). The high VHRs and laboratory experiments indicated high activity of viral lysis on their host within AD. Together, our findings may suggest that the viral community played a greater role in reducing ARGs than the proliferation, highly indicating the value of phage-based control of ARGs in AD. While we should admit that only the dsDNA virus community was evaluated, and the role of the ssDNA and RNA viral community on the spread of ARGs needs further investigation.

## Data Availability

The raw metagenomic sequence data generated in this study are deposited in NCBI with accession number of PRJNA863608.

## Supporting Information Available

metagenomic sequencing; intracellular DNA and extracellular DNA extraction; qPCR; construction of VGT and HGT network; viral contigs’ identification and clustering and taxonomic assignment; profiling of the microbial community composition; dynamics of the microbial community in AD; phage isolation and purification; lytic infectivity under the aerobic and anaerobic condition; effects of the local phage-to-bacteria ratio on the lytic infectivity; effects of pH and temperature on the lytic infectivity under anaerobic condition; difference of the antibiotic resistome among AD of the different substrate types; changes of the composition of the antibiotic resistome in AD concerning the antibiotic classes; changes of the relative abundance of selected ARGs in the samples from the batch experiment 2 in this study examined by conventional qPCR; changes of the ratio of the eARGs/tARGs during the AD of different substrates; changes of the high-risk ARGs in AD; changes of the bacterial community at the phylum level in the AD of different substrate types; ratio of ARCs in the AD experiment; PCoA based on Bray–Curtis distances showing the overall patterns of the bacterial community; heatmap showing the top 10 species in each sample; procrustes analysis showing the relationship between bacterial community at the species level and antibiotic resistome; PCoA based on Bray–Curtis distances showing the overall patterns of the viral community in AD; and Procrustes analysis showing the relationship between bacterial community at the species level and viral community in AD (PDF)
Batch experimental design; qPCR results of the selected ARGs for the samples collected from the batch experiment 2; relative abundance and the risk rank of the ARGs in each sample; basic information on the assembled contigs and annotated ARGs; taxonomy and annotation of the ARCs; relative abundance and the taxonomy information on the assembled 16S rRNA in each sample; detail information on the identified vOTUs within AD in this study; virus–host information determined by CRISPR spacer, tRNA, and homology match between viral community and binned MAGs in this study; relative abundance and the taxonomy information on the MAGs in each sample; information on the vOTUs carrying the ARGs; The virus–host information on the phages and pARBs in this study; and sequences of the identified vOTUs with ARGs in this study (XLSX)
