# Supplementary material for: Viral Communities Contribute More to the Lysis of Antibiotic-Resistant Bacteria than the Transduction of Antibiotic Resistance Genes in Anaerobic Digestion Revealed by Metagenomics
Source: Environ Sci Technol. 2024 Jan 24;58(5):2346–59. doi: 10.1021/acs.est.3c07664 (PMC10851435; doi:10.1021/acs.est.3c07664)
Supplement: Supplementary file 1 — es3c07664_si_001.pdf [file es3c07664_si_001.pdf]

Supporting Information

Viral communities contribute more to the lysis of antibiotic resistant bacteria than the transduction of antibiotic resistance genes in anaerobic digestion revealed by metagenomics

Junya Zhang[1,2,4], Tiedong Lu[5], Yunpeng Song[1,4], Ulisses Nunes da Rocha[3], Jibao Liu[1,4], Marcell Nikolausz[3], Yuansong Wei[1,4]*, Hans Hermann Richnow[2]*

[1] State Key Joint Laboratory of Environmental Simulation and Pollution Control, Research Center for Eco-Environmental Sciences, Chinese Academy of Sciences, Beijing 100085, China

[2] Department of Isotope Biogeochemistry, Helmholtz Centre for Environmental Research – UFZ, Leipzig 04318, Germany

[3] Department of Environmental Microbiology, Helmholtz Centre for Environmental Research – UFZ, 04318 Leipzig, Germany

[4] University of Chinese Academy of Sciences, Beijing 100049, China

[5] Agricultural Resource and Environment Research Institute, Guangxi Academy of Agricultural Sciences, Nanning, Guangxi, 530007, China

Author email:

Junya Zhang: jyzhang@rcees.ac.cn

Tiedong Lu: lutiedong@163.com

Yunpeng Song: leizhonghuaq0i@yeah.net

Ulisses Nunes da Rocha: ulisses.rocha@ufz.de

Jibao Liu: jbliu@rcees.ac.cn

Marcell Nikolausz: marcell.nikolausz@ufz.de

Yuansong Wei: yswei@rcees.ac.cn

Hans Hermann Richnow: hans.richnow@ufz.de

Summary: 29 pages, 11 texts, 12 graphs.

***Correspondence:**

Tel.: +86-10-62849690; Fax: +86-10-62849690;

E-mail address: **yswei@rcees.ac.cn** (Yuansong Wei); **hans.richnow@ufz.de** (Hans Hermann Richnow).

This file contains 29 pages including Text S1to S11 and Figure S1 to S12.

The Table S1 to S11 are in the Supporting tables (Additional_file_2);

The Supplementary material (Additional file 3) containing the senquence data of collected vOTU in this study has been uploaded to the Zenodo (https://doi.org/10.5281/zenodo.10279116).

Text captions

S1. Metagenomic sequencing

S2. Intracellular DNA (iDNA) and extracellular DNA (eDNA) extraction

S3. Quantitative PCR (qPCR)

S4. The construction of VGT and HGT network

S5. Viral contigs identification, clustering and taxonomic assignment

S6. Profiling of the microbial community composition

S7. Dynamics of the microbial community in AD

S8. Phage isolation and purification

S9. The lytic infectivity under the aerobic and anaerobic condition

S10. Effects of local phage-to-bacteria ratio on the lytic infectivity

S11. Effects of pH and temperature on the lytic infectivity under anaerobic condition

Figure captions

Figure S1. The difference of the antibiotic resistome among anaerobic digestion of the different substrate types

Figure S2. Changes of the composition of the antibiotic resistome in anaerobic digestion concerning the antibiotic classes.

Figure S3. Changes of the relative abundance of selected ARGs in the samples from the batch experiment 2 in this study examined by conventional qPCR.

Figure S4. Changes of the ratio of the eARGs/tARGs during the AD of different substrates.

Figure S5. Changes of the high-risk ARGs (Rank I & Rank II) in anaerobic digestion.

Figure S6. Changes of the bacterial community at the phylum level in the anaerobic digestion of different substrate types.

Figure S7. The ratio of contigs carrying ARGs (ARCs) in the anaerobic digestion experiment.

Figure S8. Principal coordinate analysis (PCoA) based on Bray-Curtis distances showing the overall patterns of the bacterial community (a); heatmap showing the top 10 species in each sample (b); Procrustes analysis showing the relationship between bacterial community at species level and antibiotic resistome (c).

Figure S9. Principal coordinate analysis (PCoA) based on Bray-Curtis distances showing the overall patterns of the viral community in anaerobic digestion.

Figure S10. Procrustes analysis showing the relationship between bacterial community at species level and viral community in anaerobic digestion.

Figure S11. The overall distribution of the lifestyle of the vOTUs identified in the anaerobic digestion.

Figure S12. The isolation of the lytic phages to the *Escherichia coli* BL21 (upper); the formed plaques of the seven purified phages under anaerobic condition (below).

Text S1. Metagenomic sequencing

These DNA samples were sent to Majorbio BioPharm Technology Co., Ltd. (Shanghai, China) for the library construction (350 bp) and pair-end sequencing (150 bp) using HiSeq 4000 platform. Briefly, DNA extract was fragmented to an average size of about 350 bp using Covaris M220 (Gene Company Limited, China) for paired-end library construction. Paired-end library was constructed using NEXTFLEX Rapid DNA-Seq (Bioo Scientific, Austin, TX, USA). Adapters containing the full complement of sequencing primer hybridization sites were ligated to the blunt-end of fragments. Paired-end sequencing was performed on Illumina HiSeq 4000 platform (Illumina Inc., San Diego, CA, USA) at Majorbio Bio-Pharm Technology Co., Ltd. (Shanghai, China) using HiSeq 4000 Reagent Kits according to the manufacturer's instructions. A total of 59 sequencing libraries were established, and approximately 788 Gb of raw data were generated, which was trimmed and quality controlled through the metaWRAP-Read_qc module by removing the reads that contained three or more ambiguous nucleotides, quality scores below 20 for more than 36 bases, or with adapter contamination [1].

Text S2. Intracellular DNA (iDNA) and extracellular DNA (eDNA) extraction

The procedure of the iDNA and eDNA extraction was referred to the previous studies with some modifications [2,3]. 4 mL of phosphate buffer (PBS) and 20 μL of proteinase K (20 mg/mL) were added to 1 mL of samples, and the mixture was kept in the water bath at 37 ºC for 30 min with a shake every 10 min. Then, 0.2 g of polyvinyl polypyrrolidone (PVPP) was added into the mixture. After shaking for 10min (250 rpm

at 25 ºC) and centrifuging for 10min (10000 g at 4 ºC), the pellets were extracted with 4 mL of PBS buffer twice. The pellets were used for iDNA extraction through the FastDNA Spin kit for soil (MP Bio), according to the manufacturer's instructions. The collected supernatants (12 mL in total) were filtered through the 0.22 μm of mixed cellulose ester membranes (47 mm diameter, Millipore, Tianjin Experiment Equipment Co. Ltd, CHN). Subsequently, the filtrate was placed on ice, an equal volume of isopropanol and 1/10 volume of sodium acetate were added to the filtrate and incubated overnight at -20 ^{o}C. After, the filtrate was centrifuged for 30min (9,600 g at 4 ºC), and then the pellets were used for the eDNA through the TIANamp Micro DNA Kit (Tiangen, China) according to the manufacturer's protocols.

Text S3.Quantitative PCR (qPCR)

Twelve typical ARGs, including *sulI*, *sulII*, *ermB*, *ermF*, *ereA*, *mefA/E*, $bla_{\text{CTX-M}}$, bla_{TEM}, *tetM*, *tetG*, *tetX* and *mcr-1* along with *intI1*, and 16S rRNA were quantified through the qPCR as our previous studies described [4–6]. Each gene was quantified in triplicate for each sample using a standard curve and a negative control. Briefly, plasmids containing these specific genes were used as standards in a 10-fold dilution and added to every qPCR run in triplicate. The 25 μL PCR reaction mixtures contained 12.5 μL of SYBR Green qPCR Super-Mix-UDG with Rox (Invitrogen, USA), 0.5 μL each of 10 mM forward and reverse primers, 10.5 μL of DNA-free water, and 1.0 mL of standard plasmid or DNA extract. The qPCR amplification was performed as follows: (1) 50 ºC, 2 min; (2) 95 ºC, 5 min; (3) 95 ºC, 20 s; (4) annealing temperature, 30 s; (5) 72 ºC, 31 s; (6) plate read, repeat steps (3) through (5) 39 more times; (7) melt-curve

analysis: 60 ºC-95 ºC, 0.2 ºC read. The reaction was conducted using an ABI Real-time PCR system 7500 (ABI, USA). The primers and annealing temperature were shown in our previous study [6]. The corresponding amplification efficiencies ranged 82.1%–100.3%.

Text S4. The construction of VGT and HGT network

The connection between ARGs and their hosts (taxonomy of the ARCs) constituted the VGT network, while HGT network indicated HGT event once happened between the connections. It was reasoned that a recent HGT event could be identified between two distantly related genomes (genera level) through the gene shared region of at least 500 bp with >99% similarity [7]. Based on this hypothesis, , if different contigs belonging to different genera shared the same ARG, we considered the HGT happened between these genera. The HGT network was further constructed to show the genus (nodes) connected by at least one observed HGT event (edges).

Text S5. Viral contigs identification, clustering and taxonomic assignment

As for VirSorter2, the potential viral contigs were first determined using the parameters (--include-groups dsDNAphage, ssDNA, NCLDV --min-score 0.5), and then the quality of each potential viral contigs was estimated with CheckV [8]. Only the contigs meet the requirements as follows were kept as viral sequences through the manual check: viral_gene >0 or viral_gene =0 AND (host_gene =0 OR score >=0.95 OR hallmark >2). As for VirFinder, the contigs with the score >=0.9 and p <=0.01 were considered as potential viral sequences, and then the contigs that met the requirements as follows were manually abandoned after the quality check with CheckV: viral_gene

=0 AND host_gene >1 or viral_gene =0 AND host_gene =1 AND length <10kb.

The valid 21,518 viral contigs were subjected to species-level clustering to create viral operational taxonomic units (vOTUs) using the ClusterGenomes scripts, following the MIUViG recommended criteria of 95% average nucleotide identity (ANI) and 85% alignment fraction (AF) [9], resulting in the identification of 13,895 vOTUs. Taxonomic assignment of these vOTUs was carried out using four methods: 1) 3827 vOTUs longer than 10 kb were protein clustered with NCBI prokaryotic viral RefSeq v201 by vConTACT2 to assign a known viral taxonomic family [10]; 2) the remaining vOTU were further assigned by majority-rules approach [11,12]. Briefly, predicted ORFs were compared to RefSeq viral proteins (release 203) using DIAMOND (-subject-cover 50, -query-cover 50, -evalue 1e-5) with bitscore ≥50. The vOTUs were assigned to a taxonomy at family level if 50% of viral proteins were assigned to the same; 3) The blastn against Integrated Microbial Genome/Virus (IMG/VR, v3.0) and RefSeq virus database (release 203) was conducted (-perc_identity 95, -evalue 1e-5) with the qcovs >75%; 4) CAT was also used to taxonomically classify the remaining vOTUs. Ultimately, 7,767 of 13,895 vOTUs (55.9%) could be assigned to a taxonomic family.

Text S6. Profiling of the microbial community composition

Microbial community composition based on the full-length 16S rRNA gene sequences assembled from the trimmed metagenomes was performed using phyloFlash (version 3.3b2) [1]. Briefly, the full-length 16S rRNA gene sequences were assembled from the 16S rRNA gene-like reads using SPAdes assembler (version 3.11.1). Then, the assembled 16s rRNA sequences collected from each sample were combined and de-

replicated using CD-HIT v4.7 at a local identity of 100%. A total of 1479 non-redundant 16s rRNA sequences with an average length of 1458 bp were finally collected. The taxonomy of these near full-length sequences of 16s rRNA was determined through blasting against the SILVA database (release 138) with the minimum identity of 90%. The RPKM (mapped reads per kilobase per million reads) of each assembled 16s rRNA sequence was calculated, while the relative abundance was determined as the ratio of the RPKM of each assembled 16s rRNA sequence in the corresponding sample.

Text S7. Dynamics of the microbial community in AD

The phylum of Firmicutes and Bacteroidota dominated in the AD, which accounted for 46.7%±12.8% and 29.1%±11.0%, respectively (Figure S6 and Table S4). The One-way PERMANOVA analysis also indicated that there existed significant difference (p<0.01) among livestock manure (CM and TM), SS and CK. The abundance of Bacteroidota in the livestock manure is generally higher than that in SS (33.4% *vs* 18.3%). The artificial substrate with higher concentration of starch also contained higher abundance of Bacteroidota, while the group with higher concentration of fatty acids showed much higher abundance of Firmicutes. The major Archaea belonged to the Halobacteriota, which accounted for 3.0%±2.9% in different substrate types.

The assembled 16s rRNA with the average sequence length of 1458 bp provided us the opportunity to look through the microbial community at the species level. Microbial community varied with substrate types as shown in the PCoA analysis at the species level (Figure 2a), and the microbial community composition was closely

associated with the inoculum sludge. The major methanogens in the non-acclimated inoculum sludge were the *Methanothrix soehngenii Gp6* no matter which substrate types (Figure 2b). *Methanothrix* is a strict acetotrophic methanogen and is associated with the stability of the methanogenesis in AD [13]. In the acclimated inoculum sludge of different substrate types, the major methanogens varied. *Methanobacterium* a hydrogenotrophic Archaea dominate the methanogens of livestock manure (PM and CM), in contrast, the *Methanosarcina flavescens*, a versatile methanogen utilizing a wide range of substrates, was the major methanogen for SS and CK [13]. Members of the genus *Syntrophomonas* acted as the major syntrophic partner of methanogens for the fermentation of long-chain (LCFA) and volatile fatty acids (VFAs) to produce acetate, which is in accordance with previous conclusion that *Syntrophomonas* were among the core essential microbial group that is present independent of the type and operation of digesters [14]. The common fermenters were the *Proteiniphilum*, which can use proteins, sugars and short chain fatty acids [14]. The *Sphaerochaeta* generally dominated in the AD of livestock manure, and *Petrimonas sp. IBARAKI* only dominated in the AD of PM, while *Petrimonas sp. B-17* dominated in the AD of CM. Nonetheless, the *Defluviitoga tunisiensis* and *Lentimicrobium* dominated in AD of SS. They were all important fermenters in the AD system.

Text S8. Phage isolation and purification

1 mL of anaerobic sludge treating domestic wastewater were collected and subjected to 15 minutes of agitation to dissociate bacteriophages; Dilute the sample 10-fold using sterile PBS buffer, followed by centrifugation at 10,000 rpm for 10 minutes

at 4°C; Employ a dual-layer agar plate method to isolate the lytic phages infecting the *Escherichia coli* BL21 (DE3) from the supernatant.

Briefly, take 100 μL of the supernatant along with 100 μL of *Escherichia coli* BL21 at logarithmic phase into a 5 mL centrifuge tube; Incubate this mixture at 37°C for 20 minutes, then add 4 mL of 50-55°C semi-solid medium of LB and gently shake to homogenize; Quickly pour the mixture onto an LB solid agar plate, allow it to solidify, and incubate at 37°C for 12-16 hours; Examine the development of phage plaques. The control was set where the supernatant is replaced with sterile PBS.

Using an inoculation loop, select phage plaques of different sizes and morphologies, and inoculate them into *Escherichia coli* BL21 at logarithmic phase; Incubate at 37°C and 180 rpm for 8-12 hours for phage re-amplification. After incubation, purify the bacteriophages using the dual-layer agar plate method for 3-5 rounds, until uniform, clear and translucent phage plaques appear on the plate, signifying successful purification.

Text S9. The lytic infectivity under the aerobic and anaerobic condition

Take 100 μL of *Escherichia coli* BL21 at logarithmic phase and add them to a 5 mL Eppendorf tube. Then, add 4 mL of 50-55°C semi-solid medium of LB and mix by shaking. Quickly pour the mixture onto an LB solid agar plate and let it solidify. Subsequently, drop 10 μL of the isolated phages onto the agar plates. Incubate the plates separately at 37°C under aerobic and anaerobic (Mitsubishi Anaerobic Culture Chamber, C-31, Japan) conditions to observe the lysis characteristics.

Text S10. Effects of local phage-to-bacteria ratio on the lytic infectivity

The lytic phage of RP1 was selected for the further analysis. Different ratios of phage-to-bacteria were prepared with the values of 0.01, 0.1, 1, 10, and 100. These mixtures were added to 5 mL of LB medium. The cultures were then incubated at 37°C with shaking at 180 rpm for 5 hours. After centrifugation at 10,000 rpm for 10 minutes, the supernatant was filtered, and the filtrate was subjected to continuous gradient dilution to determine bacteriophage titers. The value corresponding to the highest bacteriophage titer was considered the optimal phage-to-bacteria ratio for infection. Additionally, by performing serial dilution and plating, the change in the number of viable cell colonies of *Escherichia coli* BL21 was counted to determine the optimal bactericidal ratio.

The phage-to-bacteria ratio of 0.1 was considered as the optimal, where the bactericidal ratio reached over 99.94% with the highest bacteriophage titer of 7.40E+07 ± 9.90E+05 pfu/mL. We further compared the phage titer between the aerobic and anaeorbic condition. Results showed that the phage titer could reach higher value under the anaerobic condition.

Text S11. Effects of pH and temperature on the lytic infectivity under anaerobic condition

Different pH values (6, 7, 8, 9, 10) of 15 mL LB were nitrogen-flushed for 3 minutes in advance, followed by the immediate addition of 3.5 mL liquid paraffin to exclude oxygen. The lytic phage RP1 and *Escherichia coli* BL21 were added at the optimal MOI of 0.1. The cultures were incubated at 37°C without agitation for 5 hours.

After centrifugation at 10,000 rpm for 10 minutes, the cultured broth was filtered through a 0.22 μm membrane, and the filtrate was subjected to continuous gradient dilution. Bacteriophage titers were determined using the double-layer agar plate method. Additionally, serial dilution and plating were used to count changes in *E.coli* concentration.

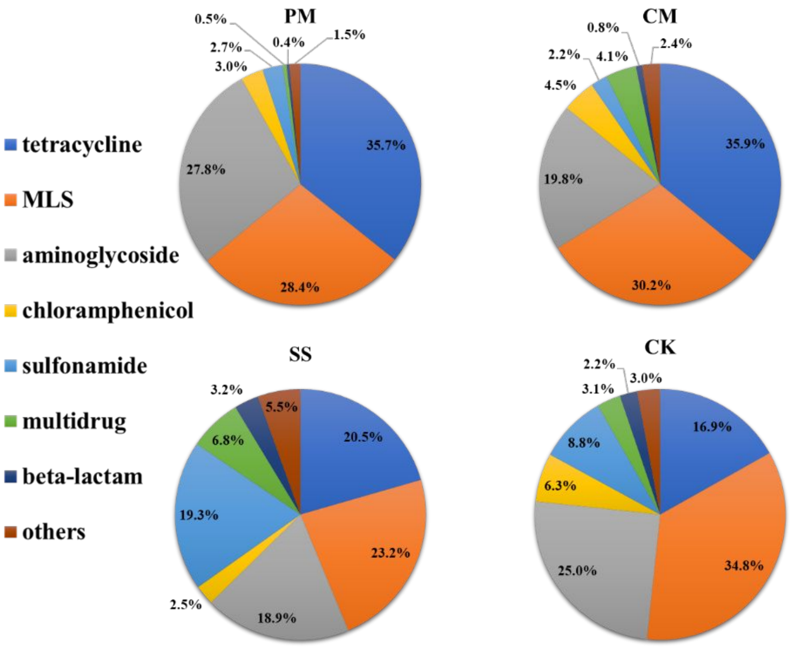

Figure S1. The difference of the antibiotic resistome among anaerobic digestion of the different substrate types: PM= pig manure, CM= chicken manure, SS= sewage sludge, CK= artificial substrates.

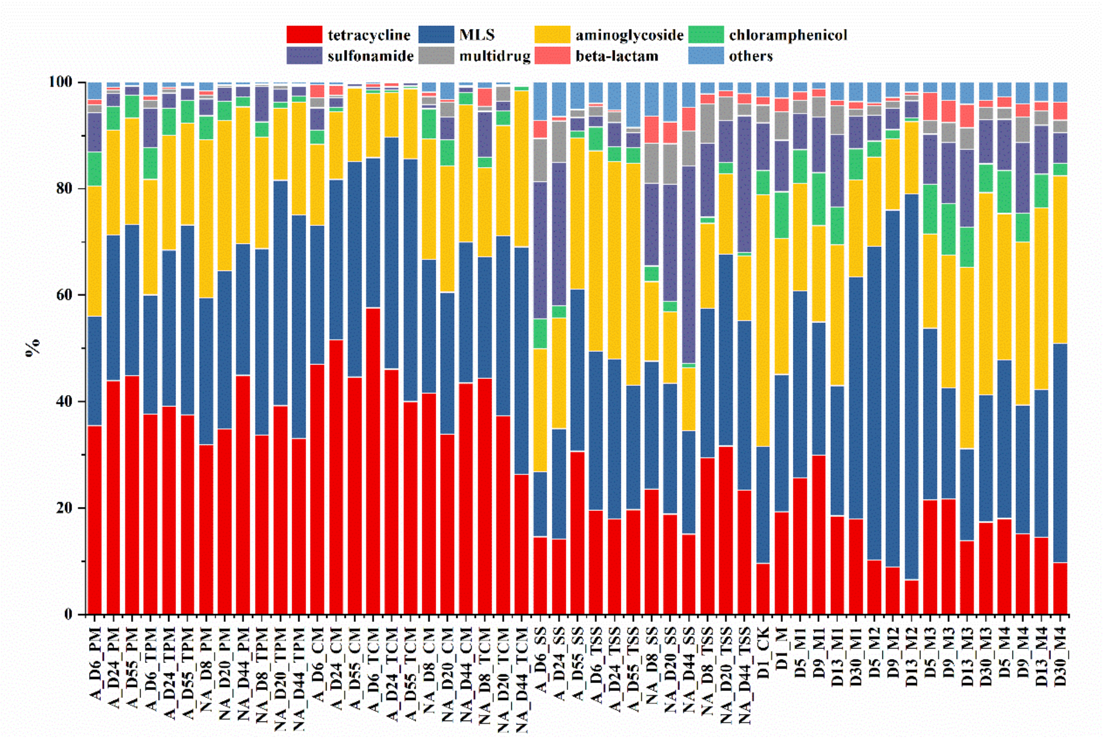

Figure S2. Changes of the composition of the antibiotic resistome in anaerobic digestion concerning the antibiotic classes. PM: pig manure; CM: chicken manure; SS: sewage sludge; CK: artificial substrates; A_ indicated the acclimated inoculum sludge; NA_ indicated the non-acclimated inoculum sludge; The M1, M2, M3, and M4 were designed to represent the substrates with balanced, high polysaccharides, high proteins and high fatty acids content, respectively.

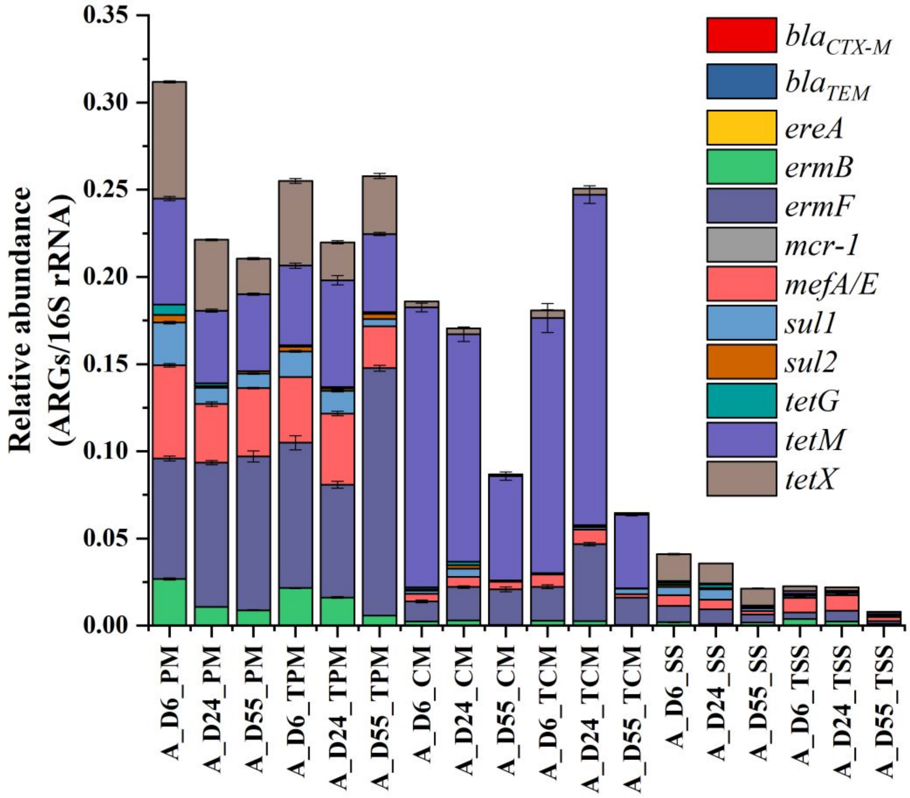

Figure S3. Changes of the relative abundance of selected ARGs in the samples from the batch experiment 2 in this study examined by conventional qPCR. PM: pig manure; CM: chicken manure; SS: sewage sludge; CK: artificial substrates; A_ indicated the acclimated inoculum sludge; NA_ indicated the non-acclimated inoculum sludge; The M1, M2, M3, and M4 were designed to represent the substrates with balanced, high polysaccharides, high proteins and high fatty acids content, respectively.

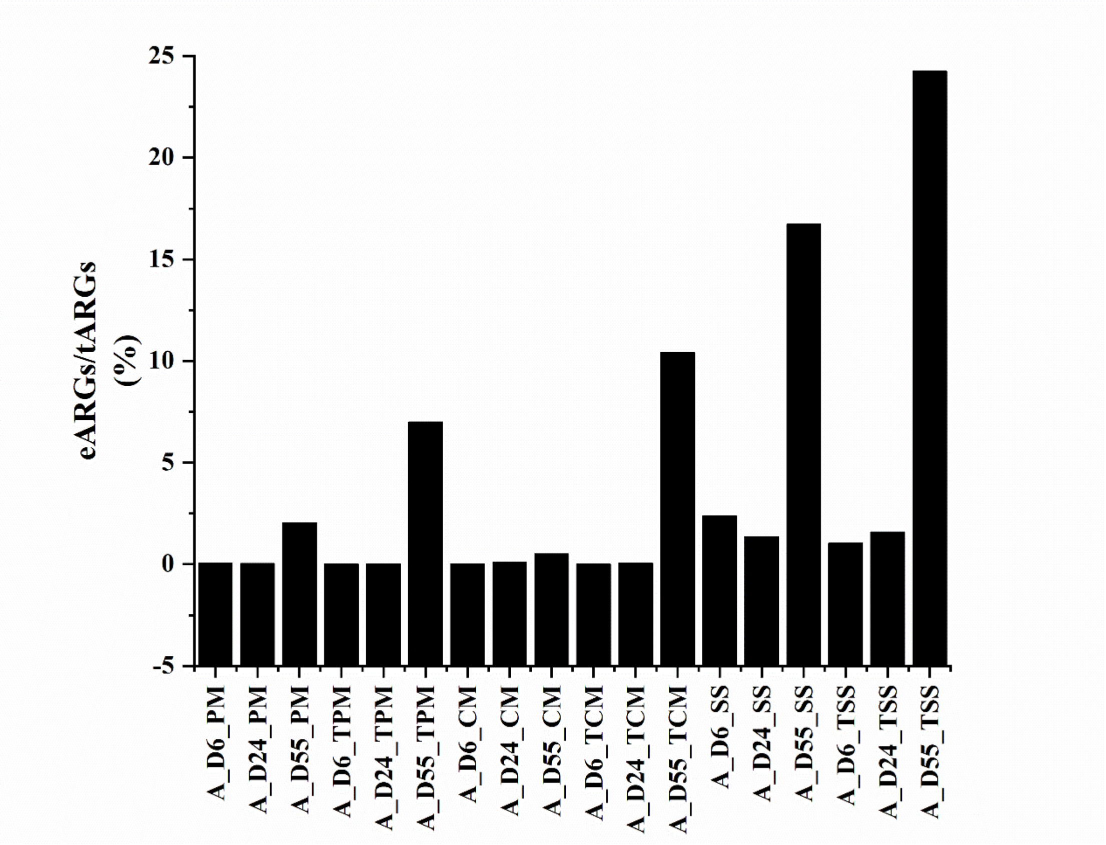

Figure S4. Changes of the ratio of the eARGs/tARGs during the AD of different substrates. PM: pig manure; CM: chicken manure; SS: sewage sludge; CK: artificial substrates; A_ indicated the acclimated inoculum sludge; NA_ indicated the non-acclimated inoculum sludge; The M1, M2, M3, and M4 were designed to represent the substrates with balanced, high polysaccharides, high proteins and high fatty acids content, respectively.

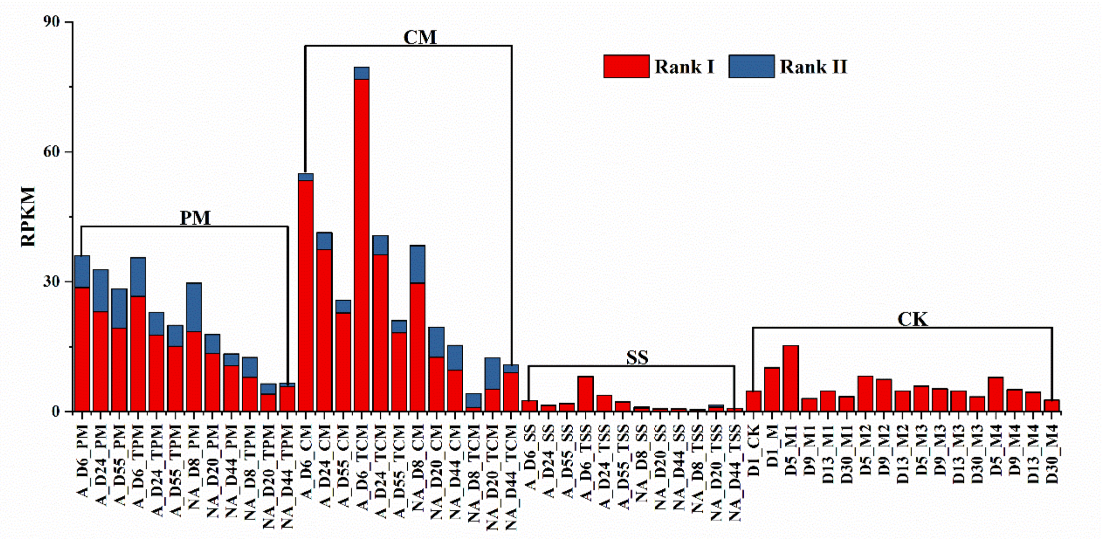

Figure S5. Changes of the high-risk ARGs (Rank I & Rank II) in anaerobic digestion. PM: pig manure; CM: chicken manure; SS: sewage sludge; CK: artificial substrates; A_ indicated the acclimated inoculum sludge; NA_ indicated the non-acclimated inoculum sludge; The M1, M2, M3, and M4 were designed to represent the substrates with balanced, high polysaccharides, high proteins and high fatty acids content, respectively.

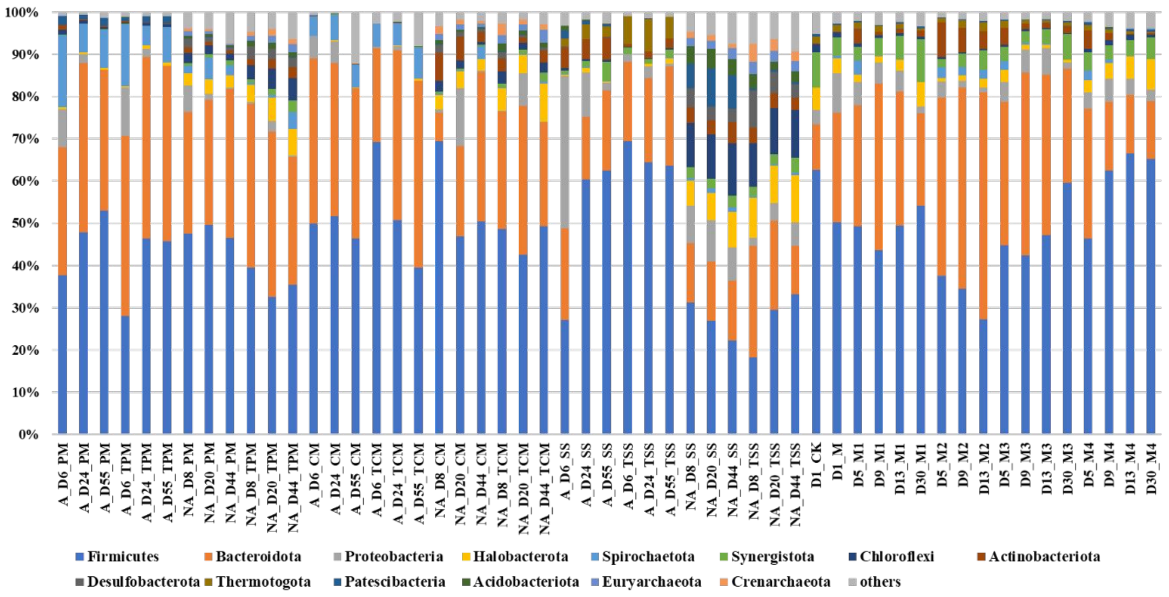

Figure S6. Changes of the bacterial community at the phylum level in the anaerobic digestion of different substrate types. PM: pig manure; CM: chicken manure; SS: sewage sludge; CK: artificial substrates; A_ indicated the acclimated inoculum sludge; NA_ indicated the non-acclimated inoculum sludge; The M1, M2, M3, and M4 were designed to represent the substrates with balanced, high polysaccharides, high proteins and high fatty acids content, respectively.

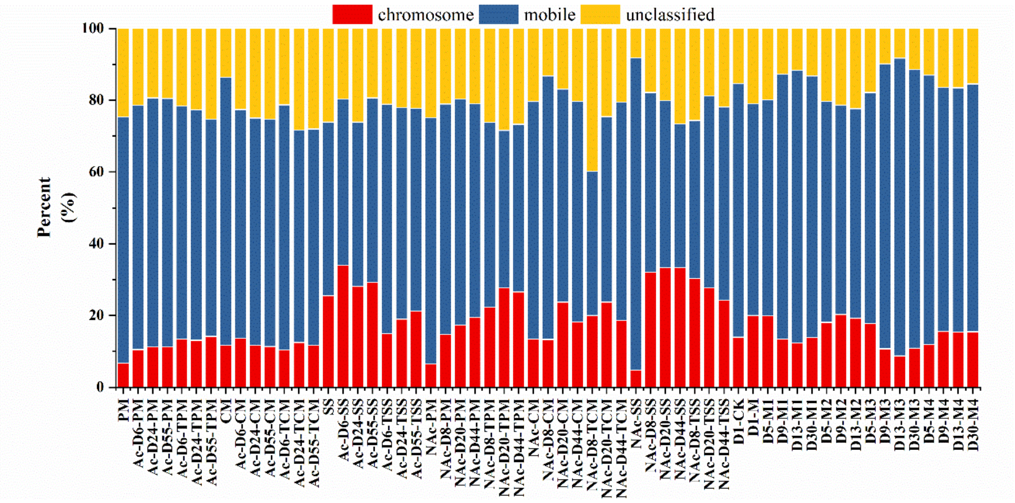

Figure S7. The ratio of contigs carrying ARGs (ARCs) in the anaerobic digestion experiment. PM: pig manure; CM: chicken manure; SS: sewage sludge; CK: artificial substrates; A_ indicated the acclimated inoculum sludge; NA_ indicated the non-acclimated inoculum sludge; The M1, M2, M3, and M4 were designed to represent the substrates with balanced, high polysaccharides, high proteins and high fatty acids content, respectively.

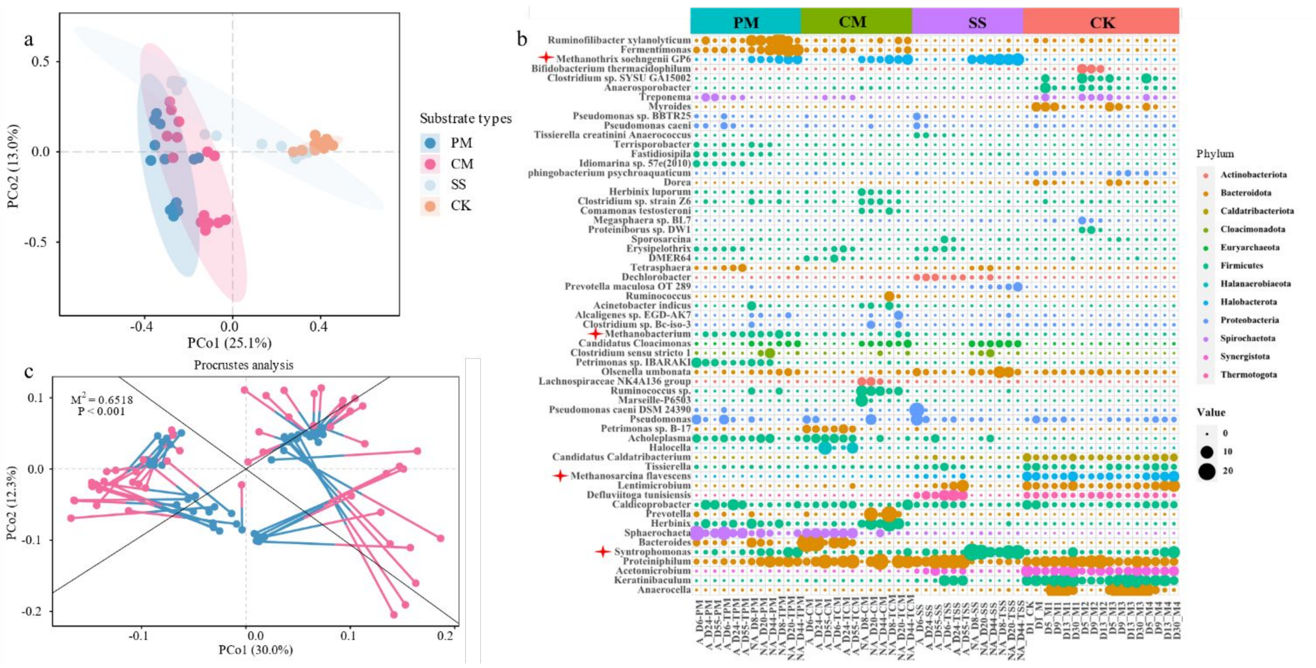

Figure S8. Principal coordinate analysis (PCoA) based on Bray-Curtis distances showing the overall patterns of the bacterial community (a); heatmap showing the top 10 species in each sample (b); Procrustes analysis showing the relationship between bacterial community at species level and antibiotic resistome (c). PM: pig manure; CM: chicken manure; SS: sewage sludge; CK: artificial substrates.

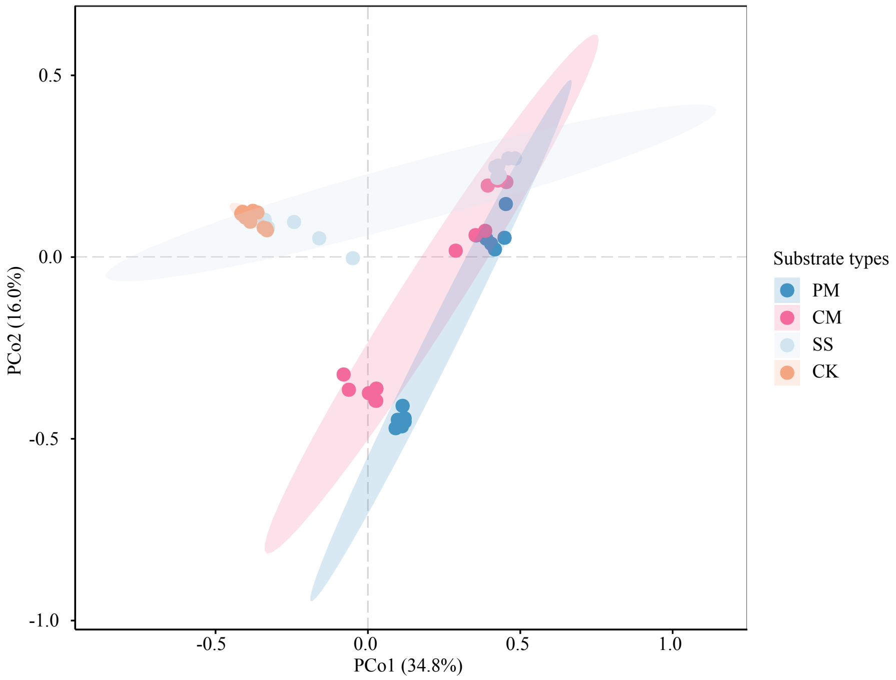

Figure S9. Principal coordinate analysis (PCoA) based on Bray-Curtis distances showing the overall patterns of the viral community in anaerobic digestion.

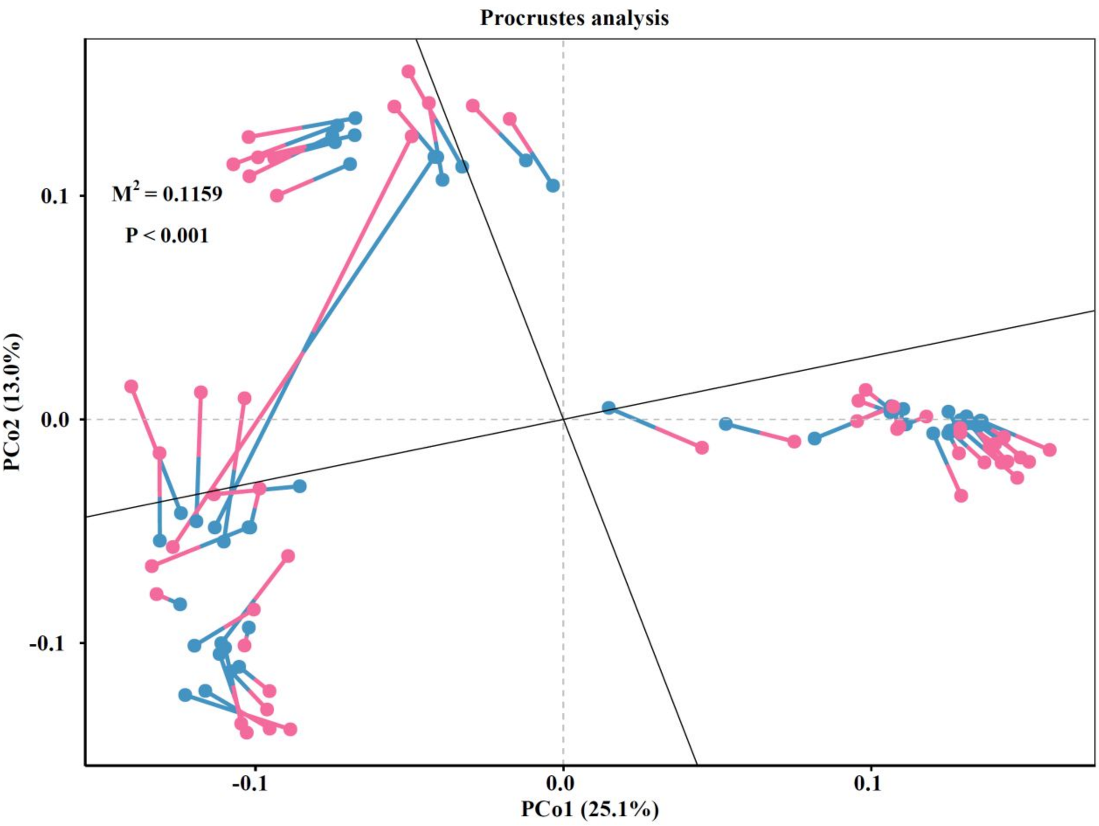

Figure S10. Procrustes analysis showing the relationship between bacterial community at species level and viral community in anaerobic digestion.

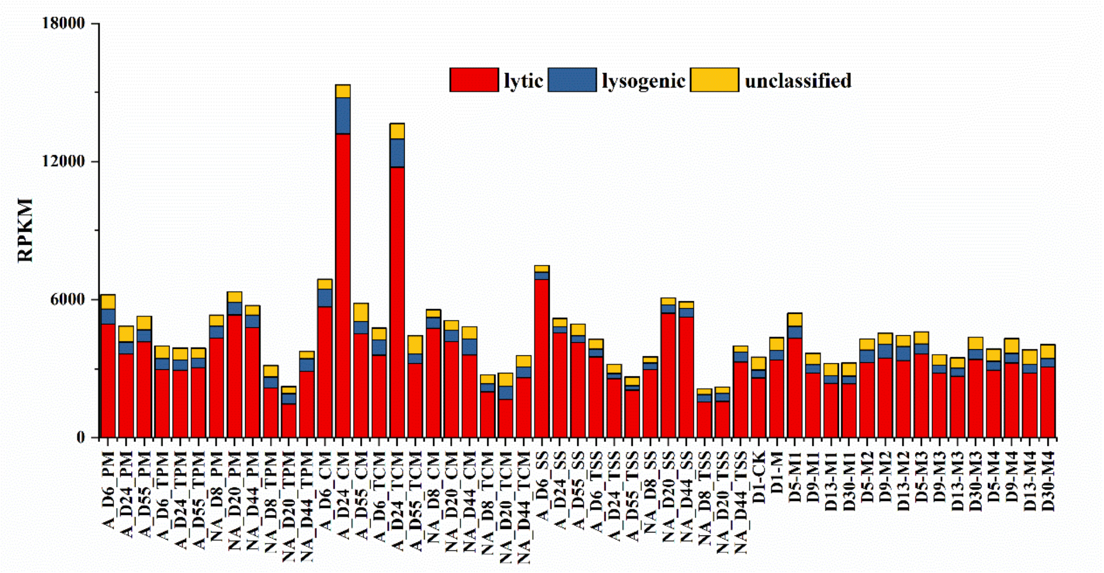

Figure S11. The overall distribution of the lifestyle of the vOTUs identified in the anaerobic digestion. For definition of codes see Table S1.

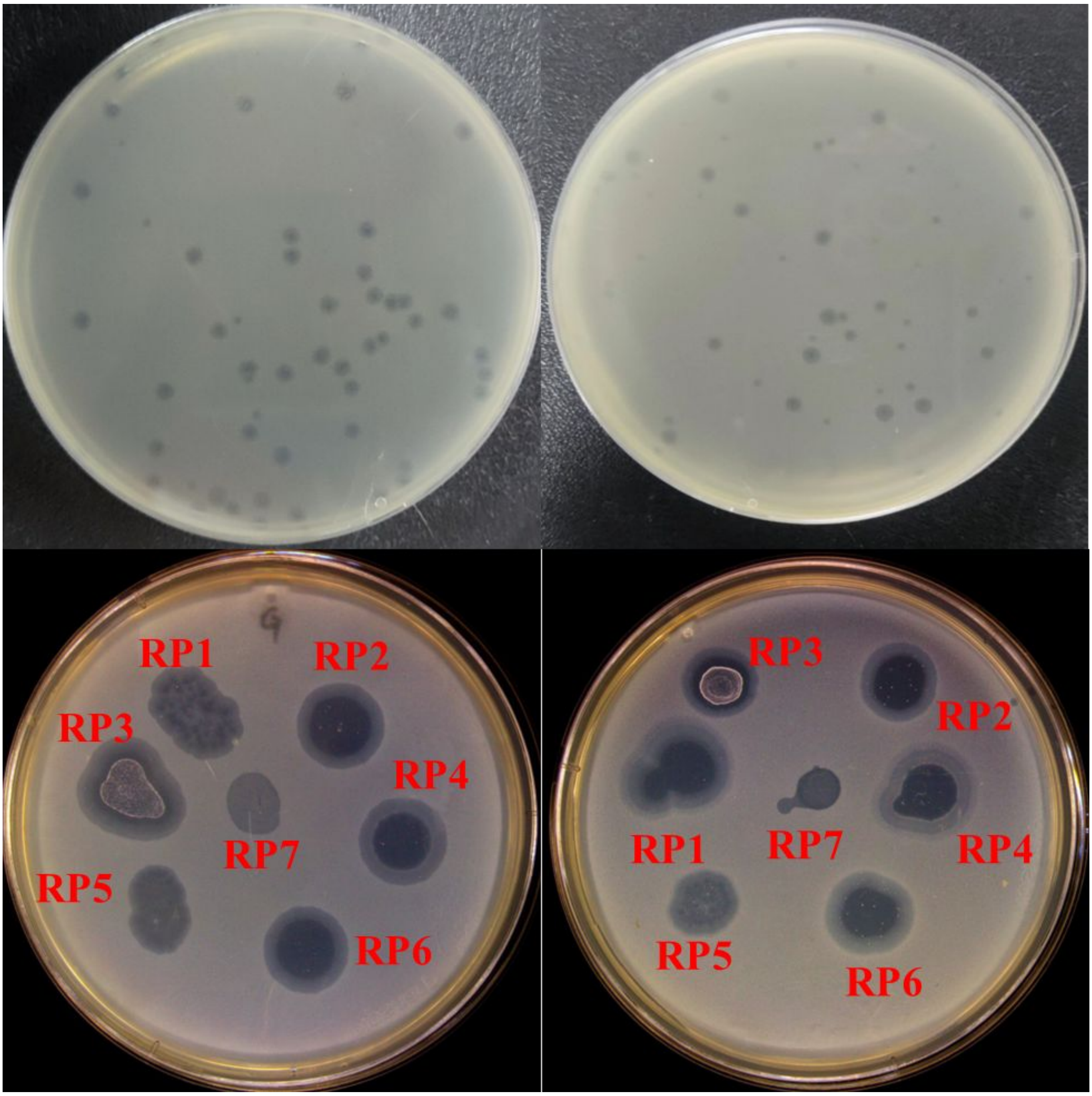

Figure S12. The isolation of the lytic phages to the *Escherichia coli* BL21 (upper); the formed plaques of the seven purified phages under anaerobic condition (below).

References

(1) Uritskiy, G. V.; Diruggiero, J.; Taylor, J. MetaWRAP - A Flexible Pipeline for Genome-Resolved Metagenomic Data Analysis. *Microbiome* **2018**, *6*, 158. https://doi.org/doi.org/10.1186/s40168-018-0541-1.

(2) Mao, D.; Luo, Y.; Mathieu, J.; Wang, Q.; Feng, L.; Mu, Q.; Feng, C.; Alvarez, P. J. J. Persistence of Extracellular DNA in River Sediment Facilitates Antibiotic Resistance Gene Propagation. *Environ. Sci. Technol.* **2014**, *48*, 71–78.

(3) Zhang, Y.; Li, X.; Snow, D. D.; Parker, D.; Zhou, Z.; Li, X. Intracellular and Extracellular Antimicrobial Resistance Genes in the Sludge of Livestock Waste Management Structures. *Environ. Sci. Technol.* **2013**, *47* (18), 10206–10213. https://doi.org/10.1021/es401964s.

(4) Zhang, J.; Yang, M.; Zhong, H.; Liu, M.; Sui, Q.; Zheng, L.; Tong, J.; Wei, Y. Deciphering the Factors Influencing the Discrepant Fate of Antibiotic Resistance Genes in Sludge and Water Phases during Municipal Wastewater Treatment. *Bioresour. Technol.* **2018**, *265* (June), 310–319. https://doi.org/10.1016/j.biortech.2018.06.021.

(5) Zhang, J.; Wang, Z.; Lu, T.; Liu, J.; Wang, Y.; Shen, P.; Wei, Y. Response and Mechanisms of the Performance and Fate of Antibiotic Resistance Genes to Nano-Magnetite during Anaerobic Digestion of Swine Manure. *J. Hazard. Mater.* **2019**, *366* (November 2018), 192–201. https://doi.org/10.1016/j.jhazmat.2018.11.106.

(6) Zhang, J.; Lu, T.; Shen, P.; Sui, Q.; Zhong, H. The Role of Substrate Types and Substrate Microbial Community on the Fate of Antibiotic Resistance Genes during Anaerobic Digestion. *Chemosphere* **2019**, *229*, 461–470. https://doi.org/10.1016/j.chemosphere.2019.05.036.

(7) Forster, S. C.; Liu, J.; Kumar, N.; Gulliver, E. L.; Gould, J. A.; Escobar-zepeda, A.; Mkandawire, T.; Pike, L. J.; Shao, Y.; Stares, M. D.; Browne, H. P.; Neville, B. A.; Lawley, T. D. Strain-Level Characterization of Broad Host Range Mobile Genetic Elements Transferring Antibiotic Resistance from the Human Microbiome. *Nat. Commun.* **2022**, *13* (2022), 1445. https://doi.org/10.1038/s41467-022-29096-9.

(8) Nayfach, S.; Camargo, A. P.; Schulz, F.; Eloe-Fadrosh, E.; Roux, S.; Kyrpides, N. C. CheckV Assesses the Quality and Completeness of Metagenome-Assembled Viral Genomes. *Nat. Biotechnol.* **2021**, *39* (5), 578–585. https://doi.org/10.1038/s41587-020-00774-7.

(9) Roux, S.; Adriaenssens, E. M.; Dutilh, B. E.; Koonin, E. V.; Kropinski, A. M.; Krupovic, M.; Kuhn, J. H.; Lavigne, R.; Brister, J. R.; Varsani, A.; Amid, C.; Aziz, R. K.; Bordenstein, S. R.; Bork, P.; Breitbart, M.; Cochrane, G. R.; Daly, R. A.; Desnues, C.; Duhaime, M. B.; Emerson, J. B.; Enault, F.; Fuhrman, J. A.; Hingamp, P.; Hugenholtz, P.; Hurwitz, B. L.; Ivanova, N. N.; Labonté, J. M.; Lee, K. B.; Malmstrom, R. R.; Martinez-garcia, M.; Mizrachi, I. K.; Ogata, H.; Páez-Espino, D.; Petit, M. A.; Putonti, C.; Rattei, T.; Reyes, A.; Rodriguez-Valera, F.; Rosario, K.; Schriml, L.; Schulz, F.; Steward, G. F.; Sullivan, M.

B.; Sunagawa, S.; Suttle, C. A.; Temperton, B.; Tringe, S. G.; Thurber, R. V.; Webster, N. S.; Whiteson, K. L.; Wilhelm, S. W.; Wommack, K. E.; Woyke, T.; Wrighton, K. C.; Yilmaz, P.; Yoshida, T.; Young, M. J.; Yutin, N.; Allen, L. Z.; Kyrpides, N. C.; Eloe-Fadrosh, E. A. Minimum Information about an Uncultivated Virus Genome (MIUVIG). *Nat. Biotechnol.* **2019**, *37* (1), 29–37. https://doi.org/10.1038/nbt.4306.

(10) Bin Jang, H.; Bolduc, B.; Zablocki, O.; Kuhn, J. H.; Roux, S.; Adriaenssens, E. M.; Brister, J. R.; Kropinski, A. M.; Krupovic, M.; Lavigne, R.; Turner, D.; Sullivan, M. B. Taxonomic Assignment of Uncultivated Prokaryotic Virus Genomes Is Enabled by Gene-Sharing Networks. *Nat. Biotechnol.* **2019**, *37* (6), 632–639. https://doi.org/10.1038/s41587-019-0100-8.

(11) Gregory, A. C.; Zablocki, O.; Zayed, A. A.; Howell, A.; Bolduc, B.; Sullivan, M. B.; Gregory, A. C.; Zablocki, O.; Zayed, A. A.; Howell, A.; Bolduc, B. Resource The Gut Virome Database Reveals Age-Dependent Patterns of Virome Diversity in the Human Gut Ll Ll Resource The Gut Virome Database Reveals Age-Dependent Patterns of Virome Diversity in the Human Gut. *Cell Host Microbe* **2020**, *28* (5), 724-740.e8. https://doi.org/10.1016/j.chom.2020.08.003.

(12) Zheng, X.; Jahn, M. T.; Sun, M.; Balcazar, J. L.; Friman, V. P.; Balcazar, J. L.; Wang, J.; Shi, Y.; Gong, X.; Hu, F.; Zhu, Y. G. Organochlorine Contamination Enriches Virus-Encoded Metabolism and Pesticide Degradation Associated Auxiliary Genes in Soil Microbiomes. *ISME J.* **2022**, *16* (5), 1397–1408.

https://doi.org/10.1038/s41396-022-01188-w.

(13) Garcia, J. Taxonomy and Ecology of Methanogens. *FEMS Microbiol. Lett.* **1990**, *87* (3–4), 297–308. https://doi.org/10.1016/0378-1097(90)90470-B.

(14) Kam, M.; Dueholm, D.; Nierychlo, M.; Andersen, K. S.; Knutsson, S.; Consortium, M. G.; Albertsen, M. MiDAS 4: A Global Catalogue of Full-Length 16S RRNA Gene Sequences and Taxonomy for Studies of Bacterial Communities in Wastewater Treatment Plants. *Nat. Commun.* **2022**, *13*, 1908. https://doi.org/10.1038/s41467-022-29438-7.
